# Redox-dependent liver gluconeogenesis impacts different intensity exercise in mice

**DOI:** 10.1038/s42255-025-01373-z

**Published:** 2025-09-18

**Authors:** Takahiro Horiuchi, Keizo Kaneko, Shinichiro Hosaka, Kenji Uno, Seitaro Tomiyama, Kei Takahashi, Maya Yamato, Akira Endo, Hiroto Sugawara, Yohei Kawana, Yoichiro Asai, Shinjiro Kodama, Junta Imai, Seiya Mizuno, Satoru Takahashi, Atsushi Takasaki, Hiraku Ono, Koutaro Yokote, Rae Maeda, Yuki Sugiura, Hideki Katagiri

**Affiliations:** 1 Department of Diabetes, Metabolism and Endocrinology, Tohoku University Graduate School of Medicine, Sendai, Japan; 2 Division of Endocrinology and Metabolism, Department of Internal Medicine, Teikyo University School of Medicine, Tokyo, Japan; 3 SiRIUS Institute of Medical Research, Tohoku University, Sendai, Japan; 4 Laboratory Animal Resource Center and Transborder Medical Research Center, Institute of Medicine, University of Tsukuba, Tsukuba, Japan; 5 Department of Endocrinology, Hematology and Gerontology, Chiba University Graduate School of Medicine, Chiba, Japan; 6 Department of Disease Prevention, Division of Clinical Preventive Medical Sciences, Center for Preventive Medical Sciences, Chiba University, Chiba, Japan; 7 Chiba University, Chiba, Japan; 8 Multiomics Platform, Center for Cancer Immunotherapy and Immunobiology, Kyoto University Graduate School of Medicine, Kyoto, Japan; 9 Human Biology Microbiome Quantum Research Center (WPI-Bio2Q), Keio University School of Medicine, Tokyo, Japan

**Keywords:** Metabolism, Carbohydrates, Biochemistry

## Abstract

Hepatic gluconeogenesis produces glucose from various substrates to meet energy demands. However, how these substrates are preferentially used under different conditions remains unclear. Here, we show that preferential supplies of lactate and glycerol modulate hepatic gluconeogenesis, thereby impacting high-intensity and low-intensity exercise capacities, respectively. We find that liver-specific knockout of phosphoenolpyruvate carboxykinase 1 (L-Pck1KO), which blocks gluconeogenesis from lactate, decreases high-intensity exercise capacity but increases low-intensity exercise capacity by enhancing gluconeogenesis from glycerol. Conversely, liver-specific knockout of glycerol kinase (L-GykKO), which inhibits glycerol-derived gluconeogenesis, induces the opposite effects by enhancing gluconeogenesis from lactate. Given that these compensatory steps depend on NAD^+^-mediated oxidation in the cytosol, we hepatically expressed NADH oxidase from *Lactobacillus brevis* (LbNOX) to decrease the cytosolic [NADH]/[NAD^+^] ratio. We find that hepatic LbNOX expression enhances gluconeogenesis from both redox-dependent substrates and increases exercise capacities at both intensities. Importantly, LbNOX-induced enhancement of high-intensity and low-intensity exercise capacities is abolished in L-Pck1KO and L-GykKO mice, respectively. Therefore, supplies of gluconeogenic substrates and cytosolic redox states, rather than altered enzyme expressions, modulate hepatic gluconeogenesis and exercise capacity at different intensities. Globally, this study shows that regulating hepatic gluconeogenesis through cytosolic redox states is a potent strategy for increasing exercise performance.

## Abstract (web summary)

This study shows that the preferential use of gluconeogenic pathways in the liver depends on exercise load.

## Main

The liver continuously produces glucose to meet the dramatically changing energy demands of daily activities. Exercise is one of the largest metabolic challenges, wherein muscle adenosine triphosphate (ATP) turnover rates can exceed those at rest by 100-fold^1^. During exercise, hepatic gluconeogenesis is highly upregulated, supported by enhanced supplies of gluconeogenic substrates, including lactate and glycerol^2–4^. Muscle glycolysis, the source of lactate, is highly enhanced in high-intensity exercise where rapid ATP production is required^1,5,6^. Meanwhile, adipose lipolysis, the source of glycerol, is more activated in low-intensity than in high-intensity exercise^7,8^. Although the primary gluconeogenic substrate seems to change depending on exercise intensities, how these gluconeogenic substrates are differentially utilized remains elusive. Here, we aimed to elucidate the physiological importance of hepatic gluconeogenesis utilizing each gluconeogenic substrate during exercise of different intensities.

**Extended Data Fig. 1:**
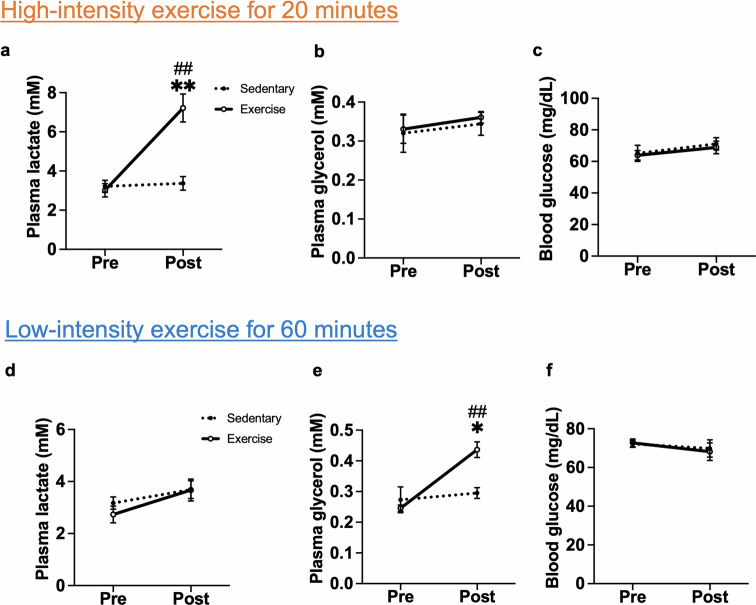
High-intensity exercise increases plasma lactate levels, while low-intensity exercise increases plasma glycerol levels. (**a**–**c)** Concentrations of plasma lactate (**a**), plasma glycerol (**b**) and blood glucose (**c**) in male C57BL/6 J wild-type mice before and after 20 min of high-intensity exercise. n = 7 per group; repeated measures two-way ANOVA followed by Holm–Šídák post hoc analysis (two-sided); (**a**) **P = 0.0036 vs Sedentary (Post), ^##^*P* = 0.0031 vs Pre (within Exercise). (**d**–**f)** Concentrations of plasma lactate (**d**), plasma glycerol (**e**) and blood glucose (**f**) in male C57BL/6 J wild-type mice before and after 60 min of low-intensity exercise. n = 7 per group; repeated measures two-way ANOVA followed by Holm–Šídák post hoc analysis (two-sided); (**e)** *P = 0.0218 vs Sedentary (Post), ^##^*P* = 0.0069 vs Pre (within Exercise). Experiments shown in (**a**–**c)** and (**d**–**f)** were conducted using distinct cohorts of biologically independent mice. All data are presented as means ± s.e.m. Source data

Given that blood lactate levels of wild-type mice reportedly rise at running speeds higher than 20 m min^−1^ (ref. ^9^), we conducted treadmill exercise experiments at up to 25 m min^−1^ as high-intensity and at 13 m min^−1^ as low-intensity exercise. As expected, in wild-type mice, 20 min of high-intensity exercise raised concentrations of plasma lactate but not plasma glycerol or blood glucose (Extended Data Fig. 1a–c). By contrast, 60 min of low-intensity exercise significantly increased plasma glycerol without changing plasma lactate and blood glucose levels (Extended Data Fig. 1d–f). Thus, lactate and glycerol supplies exceed their utilization in high-intensity and low-intensity exercise, respectively.

**Fig. 1:**
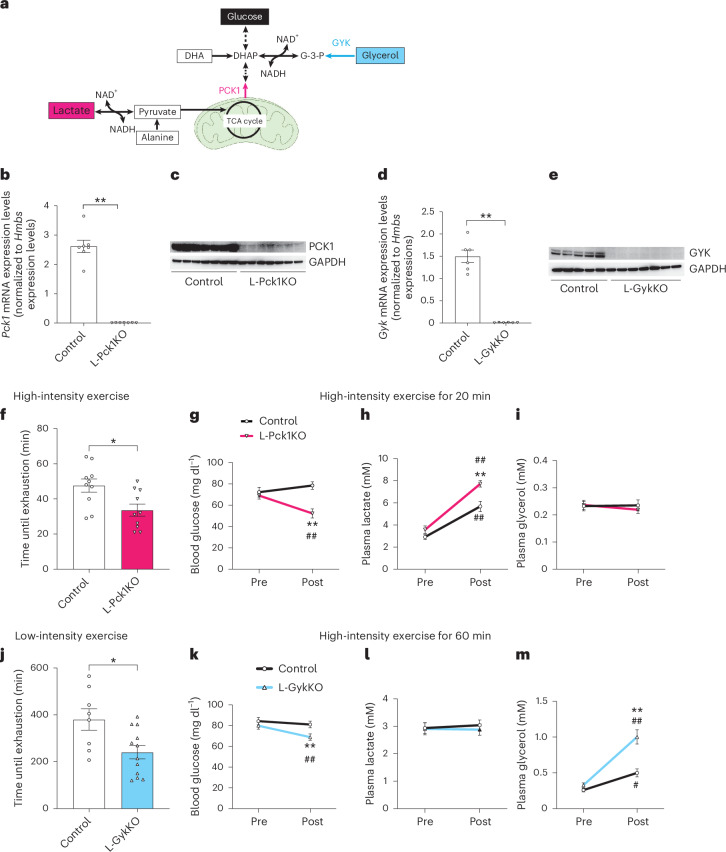
L-Pck1KO and L-GykKO decrease high-intensity and low-intensity exercise capacities, respectively. **a**, Schematic diagram of gluconeogenic pathways. Dotted arrows indicate multiple consecutive reactions, while solid arrows indicate a single reaction. The mitochondrion illustration was adapted from BioRender.com. DHAP, dihydroxyacetone phosphate; G-3-P, glycerol-3-phosphate. **b**–**e**, Expression levels of indicated mRNAs (**b**,**d**) and western blots for the indicated proteins (**c**,**e**) in the livers of male L-GykKO or L-Pck1KO mice and the corresponding controls. Signals for each target protein in **c** and **e** were obtained from different membranes that were processed in parallel. *n* = 7 (in **b**) and *n* = 6 (in **d**) per group; two-tailed unpaired *t*-test; **P < 0.0001 (in **b**); **P < 0.0001 (in **d**). **f**, Times until exhaustion in the high-intensity exercise groups of male control and L-Pck1KO mice. *n* = 10 per group; two-tailed unpaired *t*-test; *P = 0.0134. **g**–**i**, Male control and L-Pck1KO mice were subjected to 20 min of high-intensity exercise. Concentrations of blood glucose (**g**), plasma lactate (**h**) and plasma glycerol (**i**) were measured before and after exercise. *n* = 9 for control, *n* = 7 for L-Pck1KO; mixed-effects model followed by Holm–Šídák post hoc analysis (two-sided); **P = 0.0027 vs control (post), ^##^*P* = 0.0027 vs pre (within L-Pck1KO) (in **g**); **P = 0.0035 vs control (post), ^##^*P* < 0.0001 vs pre (within L-Pck1KO), ^##^*P* = 0.0001 vs pre (within control) (in **h**). **j**, Times until exhaustion in the low-intensity exercise groups of male control and L-GykKO mice. *n* = 8 for control, *n* = 12 for L-GykKO; two-tailed unpaired *t*-test; *P = 0.0138. **k**–**m**, Male control and L-GykKO mice were subjected to 60 min of low-intensity exercise. Concentrations of blood glucose (**k**), plasma lactate (**l**) and plasma glycerol (**m**) were measured before and after exercise. *n* = 12 per group; repeated measures two-way ANOVA followed by Holm–Šídák post hoc analysis (two-sided); **P = 0.0022 vs control (post), ^##^*P* = 0.0035 vs pre (within L-GykKO) (in **k**); **P = 0.0001 vs control (post), ^##^*P* < 0.0001 vs pre (within L-GykKO), ^#^*P* = 0.0204 vs pre (within control) (in **m**). Experiments shown in **b**,**c**, **d**, **e**, **f**, **g**–**i**, **j** and **k**–**m** were conducted using distinct cohorts of biologically independent mice. All data are presented as means; error bars, s.e.m. Each plot on the bar graph shows raw data. Source data

**Extended Data Fig. 2:**
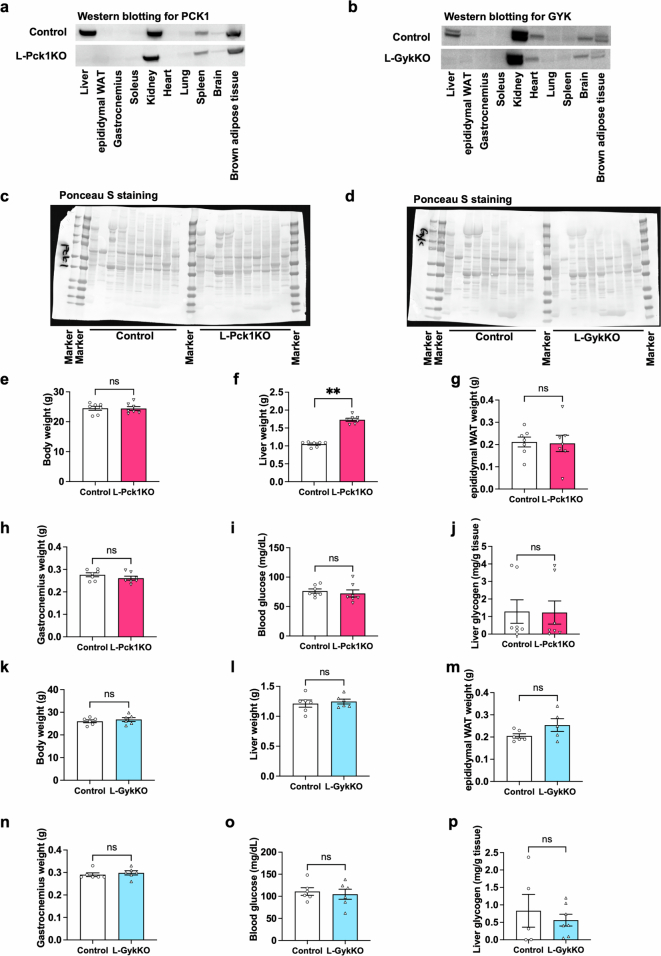
Basic phenotypes of L-Pck1KO and L-GykKO mice under steady-state conditions. (**a**,**b**) Western blotting images for PCK1 (**a**) and GYK (**b**) in the indicated tissues after tamoxifen treatment are shown. The signals shown in (**a**) and (**b**) were each obtained from the same membrane within the respective panels. (**c**,**d**) The membranes used for western blotting were stained with Ponceau S to verify that the amounts of proteins loaded in the lane differed minimally between the same tissues from male mice with the indicated genotypes. (**e**–**p**) Weights of the whole body (**e**,**k**), liver (**f**,**l**), epididymal white adipose tissue (WAT) (**g**,**m**) and gastrocnemius (**h**,**n**), as well as blood glucose levels (**i**,**o**) and liver glycogen contents (**j**,**p**) of male mice with the indicated genotypes under steady-state conditions. (**e**–**j**) n = 7 per group; (**k**–**o**) n = 6 per group (except for L-GykKO in (**m**), where n = 5 due to missing data); (**p**) n = 5 for Control, n = 7 for L-GykKO; two-tailed unpaired *t*-test; (**f**) **P < 0.0001. Experiments shown in (**a**,**c**), (**b**,**d**), (**e**–**j**), (**k**–**o**) and (**p**) were conducted using distinct cohorts of biologically independent mice. All data are presented as means ± s.e.m. Each plot on the bar graph shows raw data. Source data

Phosphoenolpyruvate carboxykinase 1 (PCK1) and glycerol kinase (GYK) are the key enzymes for gluconeogenic pathways utilizing lactate and glycerol, respectively (Fig. 1a). To separately block hepatic gluconeogenesis from each substrate, we generated mice with tamoxifen-inducible liver-specific PCK1 knockout (L-Pck1KO) and GYK knockout (L-GykKO). After tamoxifen treatment, the respective target genes and proteins were barely detectable in each of the knockout mouse livers (Fig. 1b–e), with minimally altered expressions in other organs and tissues (Extended Data Fig. 2a–d). Except for the greater liver weights in L-Pck1KO mice, as previously reported^10^, body compositions, blood glucose and liver glycogen contents in both knockout mice were similar to those of the corresponding controls (Extended Data Fig. 2e–p). Therefore, the effects of L-Pck1KO and L-GykKO were not enough to change blood glucose levels under sedentary conditions.

**Extended Data Fig. 3:**
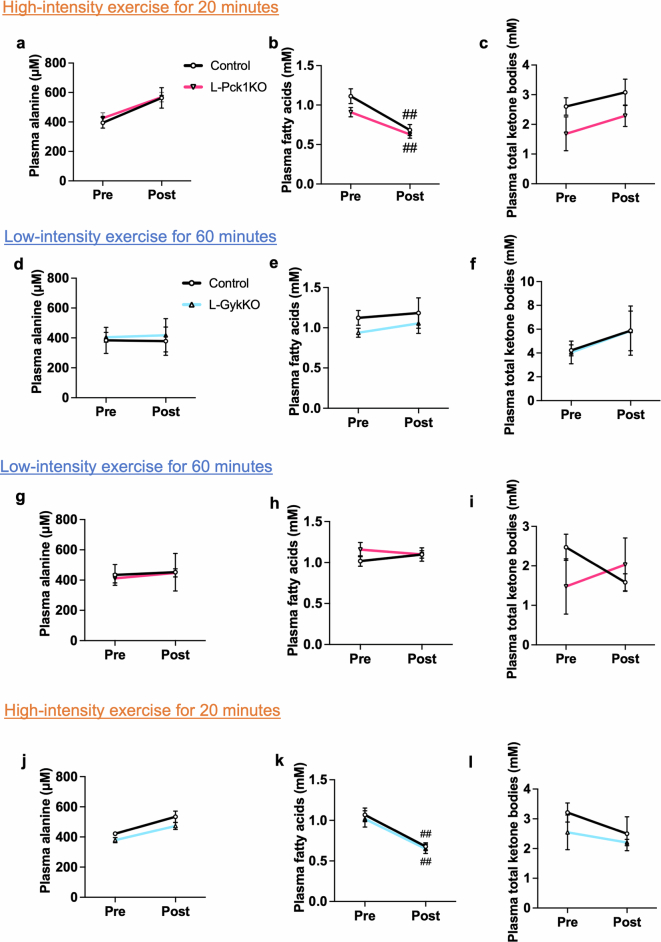
L-Pck1KO and L-GykKO do not affect plasma concentrations of alanine, fatty acids, or ketone bodies during high-intensity and low-intensity exercise. (**a**–**l**) Plasma concentrations of alanine (**a**,**d**,**g**,**j**), fatty acids (**b**,**e**,**h**,**k**), and total ketone bodies (**c**,**f**,**i**,**l**) of male L-Pck1KO or L-GykKO mice in comparison with the corresponding controls were measured before and after the indicated type of exercise. (**a**,**d**,**j**) n = 8 per group; (**b**) n = 9 for Control, n = 7 for L-Pck1KO; (**c**) n = 7 for Control, n = 5 for L-Pck1KO; (**e**) n = 9 for Control, n = 10 for L-GykKO; (**f**) n = 8 for Control, n = 7 for L-GykKO; (**g**) n = 7 for Control, n = 8 for L-Pck1KO; (**h**) n = 12 per group; (**i**) n = 7 for Control, n = 5 for L-Pck1KO; (**k**) n = 6 for Control, n = 9 for L-GykKO; (**l**) n = 7 for Control, n = 5 for L-GykKO; mixed-effects model (**b**,**c**,**e**–**g**,**i**,**k**,**l**) or repeated measures two-way ANOVA (**a**,**d**,**j**,**h**) followed by Holm–Šídák post hoc analysis (two-sided); (**b**) ^##^*P* < 0.0001 vs Pre (within Control), ^##^*P* = 0.0002 vs Pre (within L-Pck1KO); (**k**) ^##^*P* < 0.0001 vs Pre (within both Control and L-GykKO groups). All experiments shown in (**a**–**l**) were conducted using distinct cohorts of biologically independent mice. Data are presented as means ± s.e.m. Source data

By contrast, L-Pck1KO and L-GykKO mice showed decreased exercise capacities for high-intensity and low-intensity exercise, respectively (Fig. 1f,j). To ensure comparability, we fixed the exercise durations to 20 min and 60 min for high-intensity and low-intensity exercise, respectively, when none of the mice stopped exercising. L-Pck1KO and L-GykKO mice after high-intensity and low-intensity exercise showed decreased blood glucose with increased plasma lactate and glycerol levels, respectively, whereas other energy sources (alanine, fatty acids and ketone bodies) were unchanged compared to those of control mice (Fig. 1g–i,k–m and Extended Data Fig. 3a–f). These observations probably reflect the inability of PCK1-deficient and GYK-deficient hepatocytes to use lactate and glycerol, respectively, for gluconeogenesis. Therefore, hepatic gluconeogenesis from lactate via PCK1 and glycerol via GYK serves as a major energy source during high-intensity and low-intensity exercise, respectively.

**Fig. 2:**
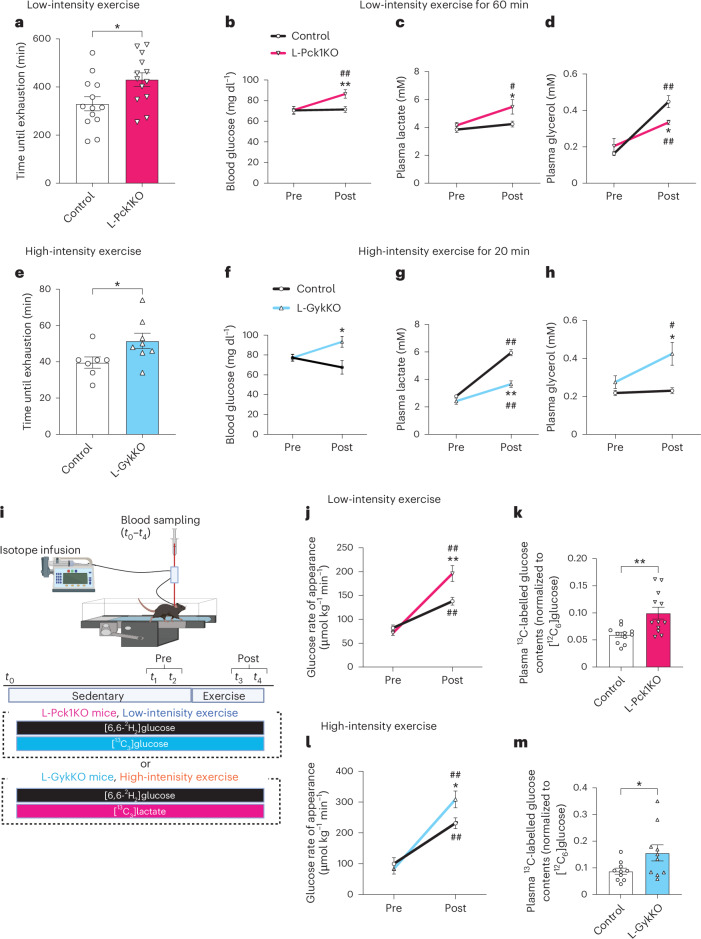
L-Pck1KO and L-GykKO increase low-intensity and high-intensity exercise capacities with enhanced gluconeogenesis from glycerol and lactate, respectively. **a**, Times until exhaustion in the low-intensity exercise groups of male control and L-Pck1KO mice. *n* = 13 per group; two-tailed unpaired *t*-test; *P = 0.0232. **b**–**d**, Male control and L-Pck1KO mice were subjected to 60 min of low-intensity exercise. Concentrations of blood glucose (**b**), plasma lactate (**c**) and plasma glycerol (**d**) were measured before and after exercise. *n* = 9 (in **b**) and 12 (in **c** and **d**) per group; repeated measures two-way ANOVA followed by Holm–Šídák post hoc analysis (two-sided); **P = 0.0089 vs control (post), ^##^*P* = 0.0089 vs pre (within L-Pck1KO) (in **b**); *P = 0.0364 vs control (post), ^#^*P* = 0.0328 vs pre (within L-Pck1KO) (in **c**); *P = 0.0145 vs control (post), ^##^*P* = 0.0097 vs pre (within L-Pck1KO), ^##^*P* < 0.0001 vs pre (within control) (in **d**). **e**, Times until exhaustion in the high-intensity exercise groups of male control and L-GykKO mice. *n* = 7 for control, *n* = 8 for L-GykKO; two-tailed unpaired *t*-test; *P = 0.0458. **f**–**h**, Male control and L-GykKO mice were subjected to 20 min of high-intensity exercise. Concentrations of blood glucose (**f**), plasma lactate (**g**) and plasma glycerol (**h**) were measured before and after exercise. *n* = 8 per group (in **f**); *n* = 6 for control, *n* = 9 for L-GykKO (in **g** and **h**); repeated measures two-way ANOVA (in **f**) or mixed-effects model (in **g** and **h**) followed by Holm–Šídák post hoc analysis (two-sided); *P = 0.0357 vs control (post) (in **f**); **P < 0.0001 vs control (post), ^##^*P* = 0.0011 vs pre (within L-GykKO), ^##^*P* < 0.0001 vs pre (within control) (in **g**); *P = 0.0307 vs control (post), ^#^*P* = 0.0159 vs pre (within L-GykKO) (in **h**). **i**, Schematic representation of isotopic metabolic flux analysis during exercise. The detailed protocols are described in the Methods. Male control and L-Pck1KO mice received prime and continuous infusions of [6,6-^2^H_2_]glucose and [^13^C_3_]glycerol starting 210 min before low-intensity exercise. Male control and L-GykKO mice received prime and continuous infusions of [6,6-^2^H_2_]glucose and [^13^C_3_]lactate starting 210 min before high-intensity exercise. Blood was collected at times *t*_0–5_. After correcting isotopic enrichment with naturally occurring isotopes from *t*_0_, metabolic flux under sedentary conditions was calculated using data from *t*_1_ and *t*_2_, while metabolic flux during exercise was determined by *t*_3_ and *t*_4_. The schematic diagram was created in BioRender.com. **j**,**l**, The glucose rate of appearance (Ra) was estimated before and during low-intensity exercise in control and L-Pck1KO mice (**j**) or high-intensity exercise in control and L-GykKO mice (**l**). *n* = 11 for control, *n* = 12 for L-Pck1KO (in **j**); *n* = 10 per group (in **l**); mixed-effects model (in **j**) or repeated measures two-way ANOVA (in **l**) followed by Holm–Šídák post hoc analysis (two-sided); **P = 0.0060 vs control (post), ^##^*P* < 0.0001 vs pre (within L-Pck1KO), ^##^*P* = 0.0060 vs pre (within control) (in **j**); *P = 0.0407 vs control (post), ^##^*P* < 0.0001 vs pre (within L-GykKO), ^##^*P* = 0.0031 vs pre (within control) (in **l**). **k**,**m**, Plasma contents of ^13^C-labelled glucose with normalization to [^12^C_6_]glucose were calculated during low-intensity exercise in control and L-Pck1KO mice (**k**) or high-intensity exercise in control and L-GykKO mice (**m**). *n* = 11 for control, *n* = 12 for L-Pck1KO (in **k**); *n* = 10 per group (in **m**); two-tailed unpaired *t*-test; *P = 0.0040 (in **k**); *P = 0.0074 (in **m**). Experiments shown in **a**, **b**, **c**,**d**, **e**, **f**, **g**,**h**, **j**,**k** and **l**,**m** were conducted using distinct cohorts of biologically independent mice. All data are presented as means; error bars, s.e.m. Each plot on the bar graph shows raw data. Source data

Surprisingly, L-Pck1KO significantly increased low-intensity exercise capacities by approximately 100 min on average (Fig. 2a). After 60 min of low-intensity exercise, L-Pck1KO mice exhibited increased blood glucose levels with less of a rise in plasma glycerol and no changes in other energy sources compared to control mice (Fig. 2b,d and Extended Data Fig. 3g–i). These results indicate that L-Pck1KO reciprocally enhanced gluconeogenesis from glycerol during low-intensity exercise. Again, intriguingly, L-GykKO increased high-intensity exercise capacities (Fig. 2e). In L-GykKO mice, 20 min of high-intensity exercise increased blood glucose levels with more modest elevations in plasma lactate and no changes in other energy sources compared to control mice (Fig. 2f,g and Extended Data Fig. 3j–l), indicating reciprocally enhanced gluconeogenesis from lactate in L-GykKO mice. Plasma lactate and glycerol in L-Pck1KO and L-GykKO mice were slightly increased after low-intensity and high-intensity exercise, respectively (Fig. 2c,h), suggesting an inability to use slightly increased supplies of these substrates. Collectively, PCK1 and GYK deletion may enhance gluconeogenesis from the unblocked alternative pathway during low-intensity and high-intensity exercise, respectively.

We further evaluated the metabolic flux, under sedentary and exercised conditions, of the models with enhanced exercise capacities (that is, L-Pck1KO and L-GykKO mice during low-intensity and high-intensity exercise, respectively) (Fig. 2i). Glucose rates of appearance showed no significant difference in L-Pck1KO or L-GykKO mice compared to the corresponding controls under sedentary conditions (Fig. 2j,l). Notably, although exercise of both intensities increased glucose rates of appearance in control mice, those of knockout mice rose further (Fig. 2j,l). These results strongly corroborate our hypothesis that gluconeogenesis is upregulated in L-Pck1KO mice during low-intensity exercise and in L-GykKO mice during high-intensity exercise. Furthermore, plasma ^13^C-labelled glucose, which is derived from [^13^C_3_]glycerol and [^13^C_3_]lactate, was higher in L-Pck1KO and L-GykKO mice during low-intensity and high-intensity exercise, respectively, than in the corresponding controls (Fig. 2k,m). Therefore, L-Pck1KO and L-GykKO enhanced gluconeogenesis from glycerol during low-intensity exercise and from lactate during high-intensity exercise, respectively, leading to reciprocally increased exercise capacities.

**Fig. 3:**
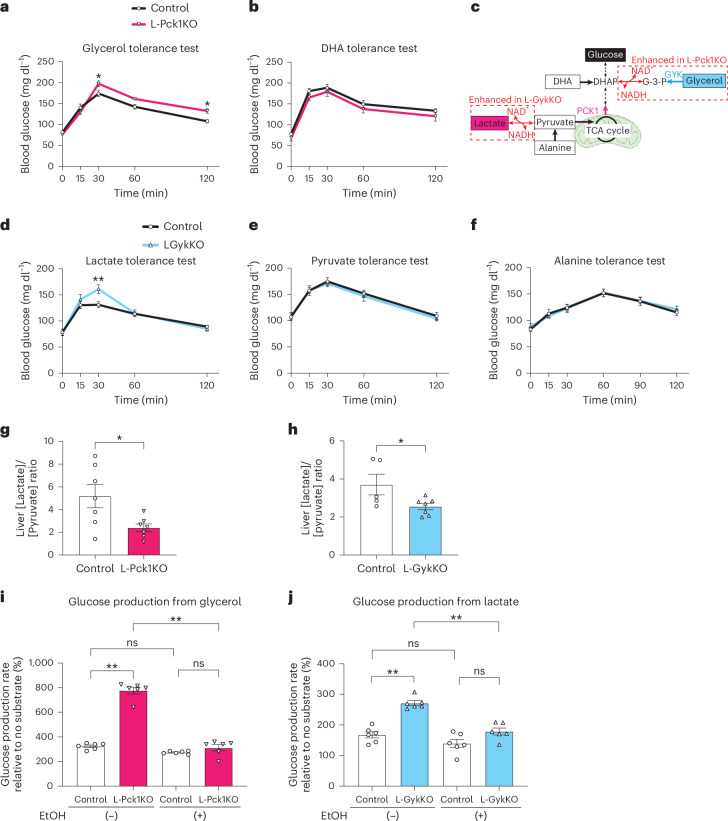
Hepatic cytosolic redox states mediate the enhanced gluconeogenesis from glycerol in L-Pck1KO mice and lactate in L-GykKO mice. **a**,**b**,**d**–**f**, Blood glucose levels of male L-Pck1KO (**a**,**b**) or L-GykKO (**d**–**f**) mice in comparison with the corresponding controls after intraperitoneal administrations of the indicated gluconeogenic substrates. *n* = 7 for control, *n* = 6 for L-Pck1KO (in **a**); *n* = 8 per group (in **b** and **d**–**f**); mixed-effects model (in **a**) or repeated measures two-way ANOVA (in **b** and **d**–**f**) followed by Holm–Šídák post hoc analysis (two-sided); *P = 0.0311 and 0.0292 vs control (at 30 and 120 min, respectively) (in **a**); **P = 0.0020 vs control (at 30 min) (in **d**). **c**, Schematic image of gluconeogenic pathways. The enhanced steps in each type of knockout mouse, suggested by the series of substrate tolerance tests, are indicated by red dotted lines. The mitochondrion illustration was adapted from BioRender.com. **g**,**h**, The liver [lactate]/[pyruvate] ratios of male L-Pck1KO mice (**g**) and L-GykKO mice (**h**) are shown in comparison to those of the corresponding controls. *n* = 7 per group (in **g**); *n* = 5 for control, *n* = 7 for L-GykKO (in **h**); two-tailed unpaired *t*-test; *P = 0.0168 (in **g**); *P = 0.0404 (in **h**). **i**,**j**, Glucose production in response to glycerol in primary hepatocytes isolated from male L-Pck1KO mice (**i**) and to lactate in primary hepatocytes isolated from male L-GykKO mice (**j**) were compared to those of the corresponding controls under the conditions with or without ethanol (EtOH) treatment. *n* = 6 per group; one-way ANOVA followed by Tukey’s post hoc analysis (two-sided); **P < 0.0001. All experiments shown in **a**–**j** were conducted using distinct cohorts of biologically independent mice. All data are presented as means; error bars, s.e.m. Each plot on the bar graph shows raw data. Source data

**Extended Data Fig. 4:**
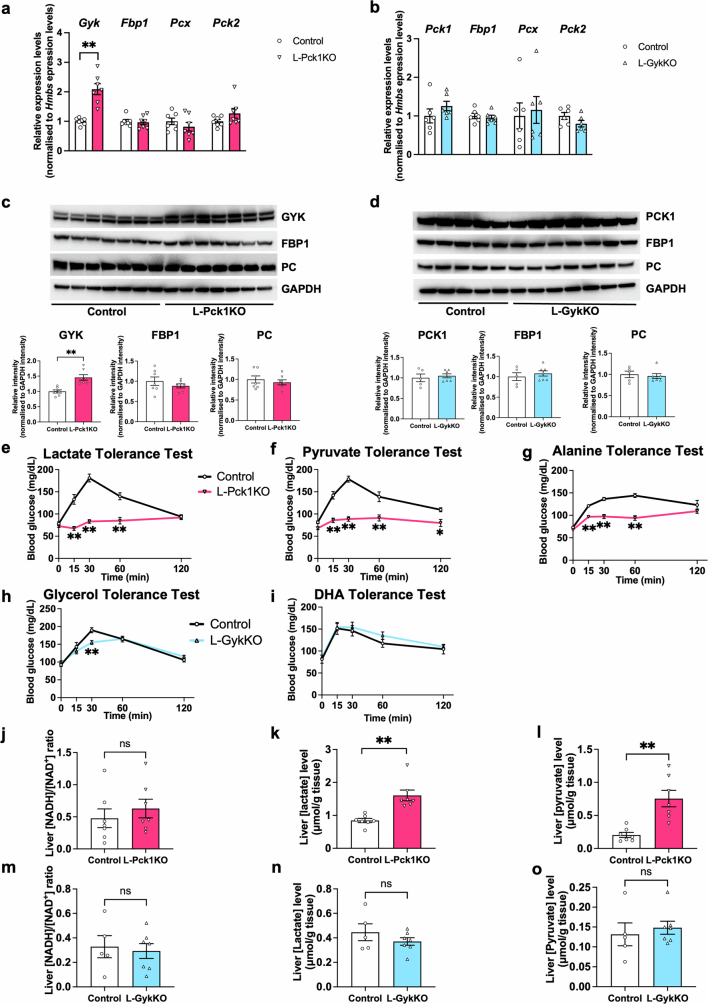
The alterations in gluconeogenic enzyme expressions and responses to gluconeogenic substrates in L-Pck1KO and L-GykKO mice. (**a**,**b**) mRNA levels of the indicated gluconeogenic genes in the livers of male L-Pck1KO (**a**) and L-GykKO (**b**) in comparison with the corresponding controls. n = 7 (**a**) and 6 (**b**) per group; two-tailed unpaired *t*-test; (**a**) **P < 0.0001 for Control vs L-Pck1KO (*Gyk* mRNA levels). (**c**,**d**) Western blots for liver gluconeogenic enzymes in male L-Pck1KO mice (**c**) and L-GykKO mice (**d**) in comparison to the corresponding controls. GAPDH was used as the loading control. Western blotting images for GAPDH in (**c**) and (**d**) correspond to those in Fig. 1c, e, respectively. Signals for each target protein in (**c**) and (**d**) were obtained from different membranes that were processed in parallel. Bar graphs represent the quantitative signal intensities of each band. (**c**) n = 7 per group; (**d**) n = 5 for Control, n = 7 for L-GykKO; two-tailed unpaired *t*-test; (**c**) **P < 0.0001 for Control vs L-Pck1KO (GYK protein levels). FBP1, fructose-1,6-bisphosphatase 1; GAPDH, glyceraldehyde-3-phosphate dehydrogenase; GYK, glycerol kinase; PC, pyruvate carboxylase; PCK1, phosphoenolpyruvate carboxykinase-1. (**e**–**i**) Blood glucose levels of male L-Pck1KO and L-GykKO mice in comparison with the corresponding controls after administrations of the indicated gluconeogenic substrates. (**e**) n = 7 for Control, n = 6 for L-Pck1KO; (**f**) n = 5 for Control, n = 7 for L-Pck1KO; (**g**) n = 5 for Control, n = 7 for L-Pck1KO; (**h**) n = 8 per group; (**i**) n = 6 per group; mixed-effects model (**e**–**g**) or repeated measures two-way ANOVA (**h**,**i**) followed by Holm–Šídák post hoc analysis (two-sided); (**e**) **P < 0.0001 vs Control (at 15, 30 and 60 min); (**f**) **P = 0.0002, < 0.0001 and = 0.0009 vs Control (at 15, 30 and 60 min, respectively), *P = 0.0250 vs Control (at 120 min); (**g**) **P = 0.0024 vs Control (at 15 min), **P < 0.0001 vs Control (at 30 and 60 min); (**h**) **P = 0.0005 vs Control (at 30 min). All experiments shown in (**e**–**i**) were conducted using distinct cohorts of biologically independent mice. (**j**–**o**) The liver [NADH]/[NAD^+^] ratios (**j**,**m**) as well as the liver contents of lactate (**k**,**n**) and pyruvate (**l**,**o**) in male mice with the indicated genotypes are shown. The results of (**k**,**l**,**n**,**o**) were used for calculating the liver [lactate]/[pyruvate] ratios shown in Fig. 3g, h. (**j**–**l**) n = 7 per group; (**m**–**o**) n = 5 for Control, n = 7 for L-GykKO; two-tailed unpaired *t*-test; (**k**) **P = 0.0009; (**l**) **P = 0.0011. Experiments shown in (**a**,**c**), (**b**), (**d**), (**e**), (**f**), (**g**), (**h**), (**i**), (**j**–**l**) and (**m**–**o**) were conducted using distinct cohorts of biologically independent mice. All data are presented as means ± s.e.m. Each plot on the bar graph shows raw data. Source data

Next, we endeavoured to elucidate the mechanism(s) underlying the reciprocal enhancements of gluconeogenesis in each type of knockout mouse. mRNA and protein expression levels of hepatic gluconeogenic enzymes were similar in each type of knockout mouse and the corresponding controls, except for increased GYK in L-Pck1KO mice (Extended Data Fig. 4a–d). We then performed a series of tolerance tests specific to various gluconeogenic substrates. As expected, compared to control mice, blood glucose levels were decreased after administering substrates requiring one of the knocked-out enzymes in their gluconeogenic pathways (lactate, pyruvate and alanine in L-Pck1KO mice, and glycerol in L-GykKO mice) (Extended Data Fig. 4e–h). L-GykKO and control mice showed similar glycaemia after administering dihydroxyacetone (DHA) (Extended Data Fig. 4i). Notably, blood glucose levels of L-Pck1KO mice were more elevated than those of control mice after administration of glycerol, but not DHA, suggesting that the step from glycerol to dihydroxyacetone phosphate was enhanced in L-Pck1KO mice (Fig. 3a–c). By contrast, L-GykKO mice showed a greater increase in glycaemia than control mice after administration of lactate, but not pyruvate or alanine, suggesting enhancement of the converting step from lactate to pyruvate in L-GykKO mice (Fig. 3c–f). Interestingly, both of the enhanced steps are redox-dependent; namely, dependent on the conversion of the oxidized form of cytosolic nicotinamide dinucleotide (NAD^+^) into its reduced form (NADH). Therefore, we reasoned that L-Pck1KO and L-GykKO had altered hepatic cytosolic redox states, which in turn enhanced gluconeogenesis from glycerol and lactate, respectively. Cytosol-specific NAD(H) concentrations were unmeasurable because of the difficulty involved with removing the major NAD(H) pool in the mitochondria and distinguishing free NAD(H) from protein-binding forms^11^. Indeed, L-Pck1KO and L-GykKO did not alter the whole-liver [NADH]/[NAD^+^] ratios (Extended Data Fig. 4j,m). We therefore measured the hepatic [lactate]/[pyruvate] ratio, a reliable and functional indicator of the cytosolic [NADH]/[NAD^+^] ratio^11–14^. In agreement with our hypothesis, hepatic [lactate]/[pyruvate] ratios in both L-Pck1KO and L-GykKO mice were significantly decreased compared to those of the corresponding controls (Fig. 3g,h and Extended Data Fig. 4j–m). Furthermore, in ex vivo experiments using isolated primary hepatocytes, the increased glucose production from glycerol in PCK1-deleted hepatocytes and from lactate in GYK-deleted hepatocytes was abrogated by ethanol treatment, which reportedly converts NAD^+^ into NADH^15,16^ (Fig. 3i,j). These findings clearly indicate that increased gluconeogenesis from redox-dependent substrates through the alternative unblocked gluconeogenic pathway is mainly caused by the decreased hepatic cytosolic [NADH]/[NAD^+^] ratios by blockade of one pathway.

**Fig. 4:**
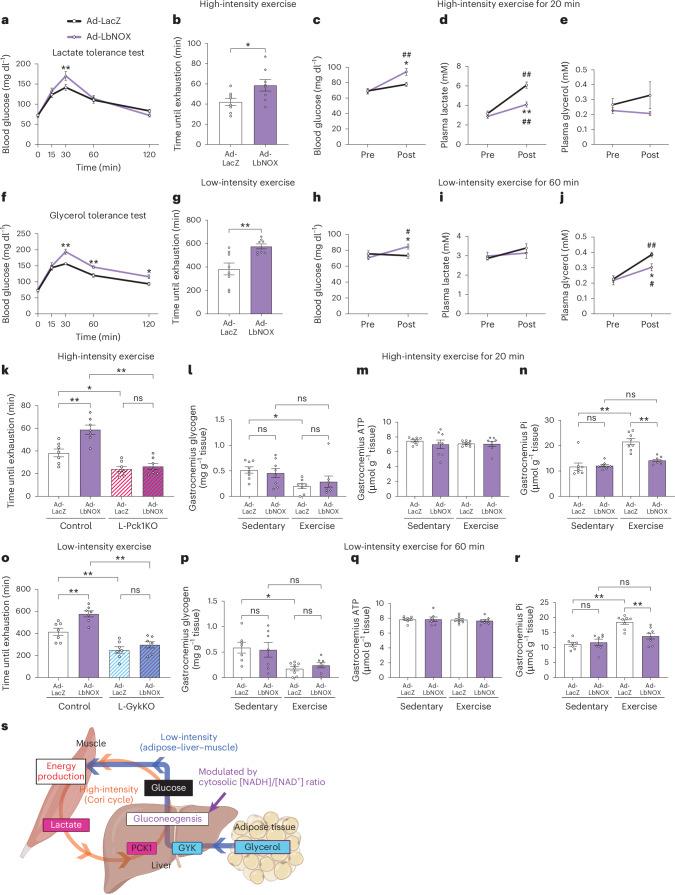
Hepatic LbNOX expression enhances capacities for exercise of both intensities by enhanced redox-dependent gluconeogenesis. **a**,**f**, Blood glucose levels of Ad-LacZ and Ad-LbNOX mice after intraperitoneal administrations of lactate (**a**) and glycerol (**f**). *n* = 8 (in **a**) and *n* = 7 (in **f**) per group; repeated measures two-way ANOVA followed by Holm–Šídák post hoc analysis (two-sided); **P < 0.0001 vs Ad-LacZ (at 30 min) (in **a**); **P = 0.0003 vs Ad-LacZ (at 30 min), **P = 0.0097 vs Ad-LacZ (at 60 min), **P = 0.0257 vs Ad-LacZ (at 120 min) (in **f**). **b**,**g**, Exercise capacities for high-intensity (**b**) and low-intensity (**g**) exercise in male Ad-LacZ and Ad-LbNOX mice were determined based on the times until exhaustion. *n* = 8 per group; two-tailed unpaired *t*-test; *P = 0.0284 (in **b**); **P = 0.0037 (in **g**). **c**–**e**,**h**–**j** Concentrations of blood glucose (**c**,**h**), plasma lactate (**d**,**i**) and plasma glycerol (**e**,**j**) in male Ad-LacZ and Ad-LbNOX mice were measured before and after 20 min of high-intensity exercise or 60 min of low-intensity exercise. *n* = 8 per group; repeated measures two-way ANOVA followed by Holm–Šídák post hoc analysis (two-sided); *P = 0.0391 vs control (post), ^##^*P* = 0.0050 vs pre (within Ad-LbNOX) (in **c**); **P < 0.0001 vs Ad-LacZ (post), ^##^*P* < 0.0001 vs pre (within both groups) (in **d**). **k**,**o**, Male control and L-Pck1KO or L-GykKO mice were intravenously administered adenovirus vector expressing LacZ or LbNOX, as indicated, 1 week after tamoxifen treatment. Then, 1 week after the adenovirus injection, mice were subjected to high-intensity (**k**) or low-intensity (**o**) exercise and their times until exhaustion were recorded. *n* = 7 per group; one-way ANOVA followed by Tukey’s post hoc analysis (two-sided); **P = 0.0008 for Ad-LacZ vs Ad-LbNOX (within control), *P = 0.0240 for control vs L-Pck1KO (within Ad-LacZ), **P < 0.0001 for control vs L-Pck1KO (within Ad-LbNOX) (in **k**); **P = 0.0037 for Ad-LacZ vs Ad-LbNOX (within control), **P = 0.0033 for control vs L-GykKO (within Ad-LacZ), **P < 0.0001 for control vs L-GykKO (within Ad-LbNOX) (in **o**). **l**–**n**,**p**–**r**, Contents of glycogen (**l**,**n**), ATP (**m**,**q**) and Pi (**n**,**r**) in the gastrocnemius were measured in male Ad-LacZ and Ad-LbNOX mice under sedentary conditions and with the indicated intensity of exercise. *n* = 8 per group (in **l**–**n**); *n* = 7 per group in sedentary, *n* = 8 per group in exercise (in **p**–**q**); one-way ANOVA followed by Tukey’s post hoc analysis (two-sided); *P = 0.0479 for sedentary vs exercise (within Ad-LacZ) (in **l**); **P < 0.0001 for sedentary vs exercise (within Ad-LacZ), **P < 0.0001 for Ad-LacZ vs Ad-LbNOX (within exercise) (in **n**); *P = 0.0170 for sedentary vs exercise (within LacZ) (in **p**); **P < 0.0001 for sedentary vs exercise (within LacZ), **P = 0.0031 for Ad-LacZ vs Ad-LbNOX (within exercise) (in **r**). **s**, Summary of the inter-organ networks during high-intensity and low-intensity exercise. In high-intensity exercise, cross-talk between muscle and liver mediated by lactate-derived gluconeogenesis has a key role in energy production. In low-intensity exercise, the inter-organ network between adipose–liver–muscle by glycerol-derived gluconeogenesis is important for energy production. The liver serves as a hub linking these inter-organ networks, wherein the hepatic cytosolic [NADH]/[NAD^+^] ratio modulates gluconeogenesis from lactate or glycerol. The schematic diagram was created in BioRender.com. Experiments shown in **a**, **b**, **c**–**e**, **f**, **g**, **h**–**j**, **k**, **l**–**n**, **o** and **p**–**r** were conducted using distinct cohorts of biologically independent mice. All data are presented as means; error bars, s.e.m. Each plot on the bar graph shows raw data. Source data

**Extended Data Fig. 5:**
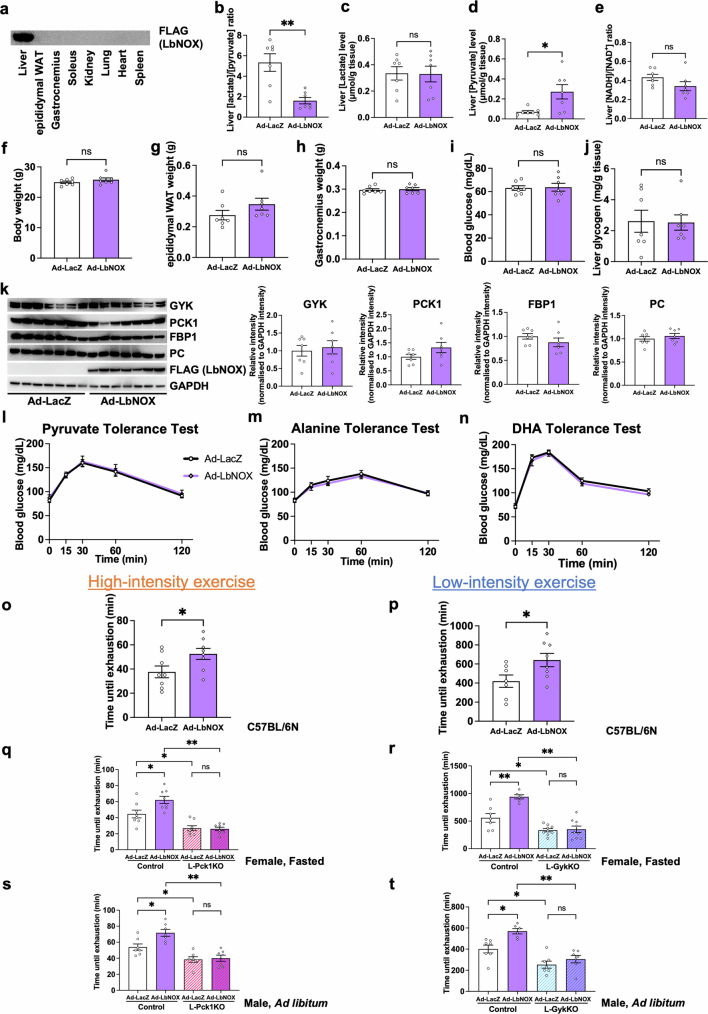
Hepatic LbNOX expression enhances capacities for exercise of both intensities via enhanced redox-dependent gluconeogenesis, even in female mice or in the fed state. (**a**) Expressions of FLAG-tagged LbNOX in the indicated tissues from a male Ad-LbNOX mouse were examined by western blotting for FLAG. (**b**–**e**) The liver [lactate]/[pyruvate] ratios (**b**), lactate (**c**), pyruvate (**d**) and the [NADH]/[NAD^+^] ratios (**e**) in male Ad-LacZ and Ad-LbNOX mice under steady-state conditions are shown. n = 7 per group; two-tailed unpaired *t*-test; (**b**) **P = 0.0015; (**d**) *P = 0.0178. (**f**–**j**) Weights of the whole body (**f**), epididymal white adipose tissue (WAT) (**g**) and gastrocnemius muscles (**h**), as well as blood glucose levels (**i**), and liver glycogen contents (**j**) were measured in male Ad-LacZ and Ad-LbNOX mice. n = 7 per group; two-tailed unpaired *t*-test. (**k**) Western blots for FLAG and the indicated gluconeogenic enzymes in the livers of male Ad-LacZ and Ad-LbNOX mice are shown. Signals for each target protein were obtained from different membranes that were processed in parallel. Bar graphs represent the quantitative signal intensities of each band. n = 7 per group; two-tailed unpaired *t*-test. FBP1, fructose-1,6-bisphosphatase 1; GAPDH, glyceraldehyde-3-phosphate dehydrogenase; GYK, glycerol kinase; PC, pyruvate carboxylase; PCK1, phosphoenolpyruvate carboxykinase-1. (**l**–**n**) Blood glucose levels of Ad-LacZ and Ad-LbNOX mice after intraperitoneal administration of pyruvate (**l**), alanine (**m**) and dihydroxyacetone (DHA) (**n**). (**l**) n = 7 for Ad-LacZ, n = 6 for Ad-LbNOX; (**m**,**n**) n = 7 per group. Mixed-effects model (**l**) or repeated measures two-way ANOVA (**m**,**n**) were performed (two-sided), without post hoc analysis due to the absence of significant interaction or group effects. (**o**,**p**) Times until exhaustion of C57BL/6 N mice administered with Ad-LacZ and Ad-LbNOX were measured in high-intensity (**o**) and low-intensity (**p**) exercise. (**o**) n = 8 per group; (**p**) n = 7 for Ad-LacZ, n = 8 for Ad-LbNOX; two-tailed unpaired *t*-test; (**o**) *P = 0.0441; (**p**) *P = 0.0372. (**q**,**r**) Times until exhaustion of female mice with the indicated genotypes with the hepatic expression of LacZ or LbNOX were measured in high-intensity (**q**) and low-intensity (**r**) exercise under fasted conditions. (**q**) n = 8 per group; (**r**) n = 7 (Control + Ad-LacZ), n = 6 (Control + Ad-LbNOX), n = 9 each (L-GykKO + Ad-LacZ and Ad-LbNOX); one-way ANOVA followed by Tukey’s post hoc analysis (two-sided); (**q**) *P = 0.0145 for Ad-LacZ vs Ad-LbNOX (within Control), *P = 0.0129 for Control vs L-Pck1KO (within Ad-LacZ), **P < 0.0001 for Control vs L-Pck1KO (within Ad-LbNOX); (**r**) **P = 0.0006 for Ad-LacZ vs Ad-LbNOX (within Control), *P = 0.0326 for Control vs L-GykKO (within Ad-LacZ), *P < 0.0001 for Control vs L-GykKO (within Ad-LbNOX). (**s**,**t**) Times until exhaustion of male mice with the indicated genotypes with the hepatic expression of LacZ or LbNOX were measured in high-intensity (**s**) and low-intensity (**t**) exercise under *ad libitum* conditions. n = 7 per group except for the Control + Ad-LbNOX group in (**t**), where n = 6; one-way ANOVA followed by Tukey’s post hoc analysis (two-sided); (**s**) *P = 0.0169 for Ad-LacZ vs Ad-LbNOX (within Control), *P = 0.0453 for Control vs L-Pck1KO (within Ad-LacZ), **P < 0.0001 for Control vs L-Pck1KO (within Ad-LbNOX); (**t**) *P = 0.0102 for Ad-LacZ vs Ad-LbNOX (within Control), *P = 0.0202 for Control vs L-GykKO (within Ad-LacZ), **P < 0.0001 for Control vs L-GykKO (within Ad-LbNOX). Experiments shown in (**a**), (**b**–**k**), (**l**), (**m**), (**n**), (**o**), (**p**), (**q**), (**r**), (**s**) and (**t**) were conducted using distinct cohorts of biologically independent mice. All data are presented as means ± s.e.m. Each plot on the bar graph shows raw data. Source data

We further investigated whether the altered hepatic cytosolic redox state is sufficient to enhance exercise capacities. To specifically lower the hepatic cytosolic [NADH]/[NAD^+^] ratio, we applied water-forming NADH oxidase from *Lactobacillus brevis* (LbNOX), which converts cytosolic NADH into NAD^+^ without affecting the mitochondrial redox state^17,18^. Using an adenoviral system, we induced LbNOX selectively in C57BL/6J mouse livers (Ad-LbNOX mice), which reduced hepatic [lactate]/[pyruvate] ratios without significant changes in the whole-liver [NADH]/[NAD^+^] ratios (Extended Data Fig. 5a–e), as previously reported^17,18^. Hepatic LbNOX expression minimally altered body compositions, liver glycogen contents and hepatic gluconeogenic enzyme expressions (Extended Data Fig. 5f–k). As expected, Ad-LbNOX mice exhibited higher blood glucose levels than control mice after administrations of redox-dependent substrates (lactate and glycerol) (Fig. 4a,f) but not redox-independent substrates (pyruvate, alanine and DHA) (Extended Data Fig. 5l–n). Notably, hepatic LbNOX expression markedly increased the capacities for exercise of both intensities (Fig. 4b,g). Hepatic LbNOX expression in C57BL/6N mice produced similar results (Extended Data Fig. 5o,p). Consistently, compared to control mice, Ad-LbNOX mice showed higher blood glucose with lower plasma lactate and similar plasma glycerol levels during high-intensity exercise or with lower plasma glycerol, and unchanged plasma lactate during low-intensity exercise (Fig. 4c–e,h–j). Therefore, decreased hepatic cytosolic [NADH]/[NAD^+^] ratios are sufficient to enhance capacities for exercise of both intensities.

Given that NAD(H) is involved in numerous enzymatic reactions, we investigated whether the LbNOX-induced increases in exercise capacities are genuinely mediated by enhanced gluconeogenesis by examining the effects of hepatic LbNOX expression in combination with L-Pck1KO or L-GykKO. Most strikingly, the LbNOX-induced enhancements of high-intensity and low-intensity exercise capacities were almost completely blocked by L-Pck1KO and L-GykKO, respectively (Fig. 4k,o). These results clearly indicate that enhanced exercise capacities in response to decreased hepatic cytosolic [NADH]/[NAD^+^] ratios are essentially attributable to enhanced gluconeogenesis from lactate and glycerol in high-intensity and low-intensity exercise, respectively. Similar results were obtained using female mice (Extended Data Fig. 5q,r). Furthermore, although most of our experiments were conducted on fasted animals to focus on gluconeogenesis, we obtained similar results even under ad libitum conditions (Extended Data Fig. 5s,t), probably because hepatic glycogen is depleted before exhaustion^19^. Therefore, gluconeogenesis is a potent determinant of exercise capacities, regardless of gender or feeding conditions. Hepatic cytosolic redox states modulate capacities for exercise with different intensities through two distinct hepatic gluconeogenic pathways.

**Extended Data Fig. 6:**
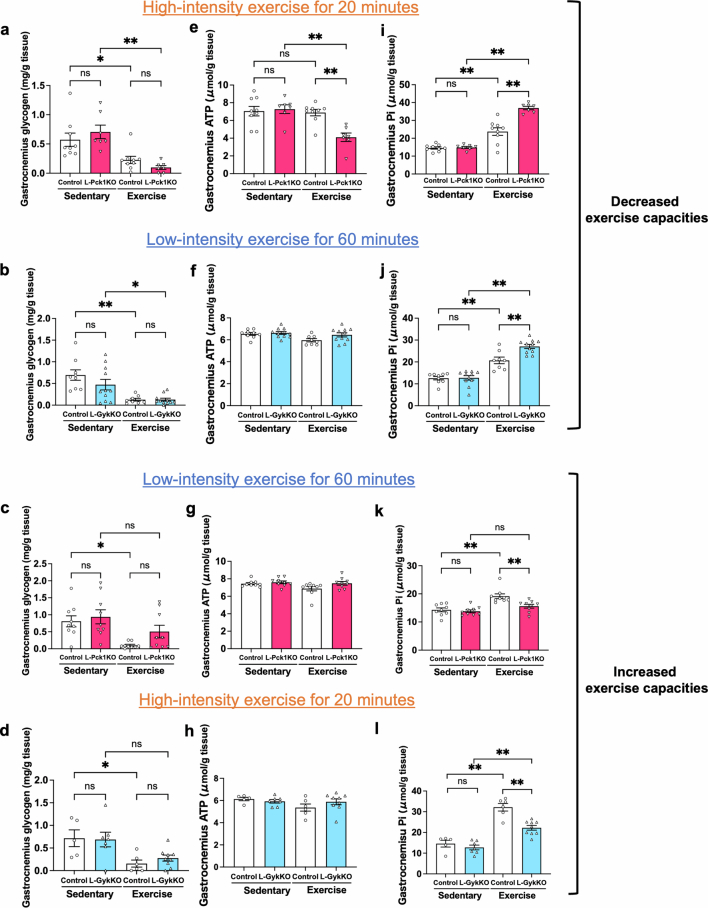
Muscle metabolite alterations during high-intensity and low-intensity exercise. (**a**–**l**) Contents of glycogen (**a**–**d**), ATP (**e**–**h**) and Pi (**i**–**l**) in the gastrocnemius were measured in male L-Pck1KO and L-GykKO mice in comparison with those of the corresponding controls under sedentary conditions and with the indicated intensity of exercise. (**a**,**e**,**i**) n = 9 for Control in both Sedentary and Exercise groups, n = 7 for L-Pck1KO in both Sedentary and Exercise groups; (**b**,**f**,**j**) n = 9 for Sedentary-Control, n = 8 for Exercise-Control, n = 11 for L-GykKO in both Sedentary and Exercise groups; (**c**,**g**,**k**) n = 9 per group; (**d**,**h**,**l**) n = 5 for Sedentary-Control, n = 7 for Sedentary-L-GykKO, n = 6 for Exercise-Control, n = 9 for Exercise-L-GykKO; one-way ANOVA followed by Tukey’s post hoc analysis (two-sided); (**a**) *P = 0.0350 for Sedentary vs Exercise (within Control), **P = 0.0006 for Sedentary vs Exercise (within L-Pck1KO); (**b**) **P = 0.0012 for Sedentary vs Exercise (within Control), *P = 0.0351 for Sedentary vs Exercise (within L-GykKO); (**c**) *P = 0.0210 for Sedentary vs Exercise (within Control); (**d**) *P = 0.0349 for Sedentary vs Exercise (within Control); (**e**) **P = 0.0007 for Sedentary vs Exercise (within L-Pck1KO), **P = 0.0015 for Control vs L-Pck1KO (within Exercise); (**i**) **P = 0.0001 for Sedentary vs Exercise (within Control), **P < 0.0001 for Sedentary vs Exercise (within L-Pck1KO) and for Control vs L-Pck1KO (within Exercise); (**j**) **P < 0.0001 for all indicated comparisons; (**l**) **P < 0.0001 for Sedentary vs Exercise (within Control), **P = 0.0002 for Sedentary vs Exercise (within L-GykKO) and for Control vs L-GykKO (within Exercise). Experiments shown in (**a**,**e**,**i**), (**b**,**f**,**j**), (**c**,**g**,**k**) and (**d**,**h**,**l**) were conducted using distinct cohorts of biologically independent mice. Data are presented as means ± s.e.m. Each plot on the bar graph shows raw data. Source data

**Extended Data Fig. 7:**
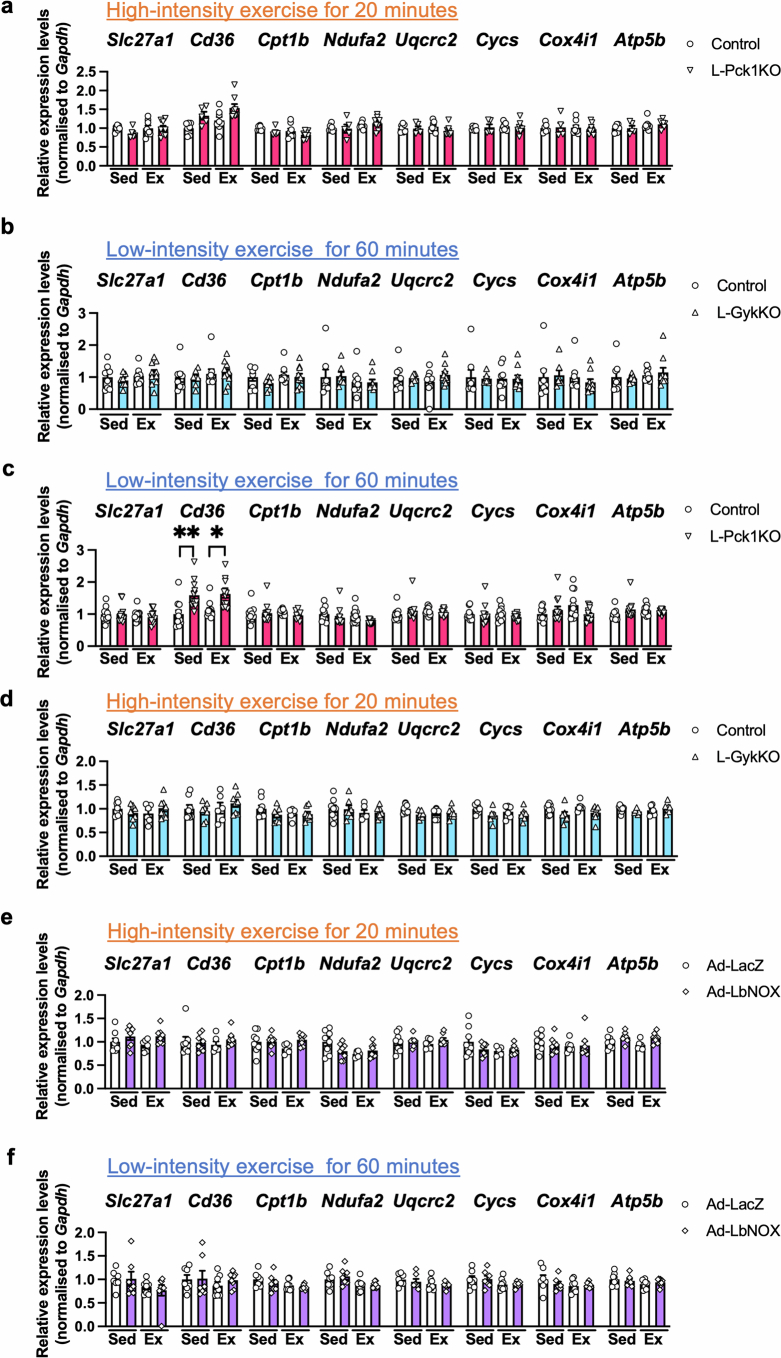
Liver-specific modulations of gluconeogenesis have minimal effects on the muscular expressions of genes related to fatty acid oxidation and the mitochondrial electron transport chain. (**a**–**f**) Expression levels of mRNA related to fatty acid oxidation and the mitochondrial electron transport chain (ETC) of male L-Pck1KO, L-GykKO and Ad-LbNOX mouse gastrocnemius, for comparison with the corresponding controls, were measured under sedentary conditions and the indicated intensity of exercise. Expression levels of mRNA were normalised to *glyceraldehyde-3-phosphate dehydrogenase* (*Gapdh*) expression levels. Sed, sedentary; Ex, exercised. (**a**) n = 7 for Sed-Control, n = 5 for Sed-L-Pck1KO, n = 8 for both Ex-Control and Ex-L-Pck1KO; (**b**) n = 8 for Sed-Control, n = 7 for Sed-L-GykKO, n = 9 for Ex-Control, n = 10 for Ex-L-GykKO; (**c**) n = 12 per group, except for Sed-Control (n = 11); (**d**) n = 8 for Sed-Control and Ex-L-GykKO, n = 7 for Sed-L-GykKO, n = 6 for Ex-Control; (**e**) n = 8 per group, except for Ex-Ad-LacZ (n = 6); (**f**) n = 7 for Sed-Ad-LacZ and Sed-Ad-LbNOX, n = 8 for Ex-Ad-LacZ and Ex-Ad-LbNOX; one-way ANOVA followed by Tukey’s post hoc analysis (two-sided); (**c**) **P = 0.0039 for Control vs L-Pck1KO (within Sed), *P = 0.0116 for Control vs L-Pck1KO (within Ex). All experiments shown in (**a**–**f**) were conducted using distinct cohorts of biologically independent mice. Data are presented as means ±  s. e. m. Each plot on the bar graph shows raw data. Source data

We further examined muscle phenotypes under sedentary and exercised conditions. As exercise progresses, muscle glycogen content decreases, and ATP breakdown products, for example, inorganic phosphate (Pi), accumulate before ATP content decreases^20–22^. Although muscle glycogen contents were reduced after exercise in the models with decreased exercise capacities (that is, L-Pck1KO mice with high-intensity exercise and L-GykKO mice with low-intensity exercise), the decrements in the models with increased exercise capacities (that is, L-Pck1KO mice with low-intensity exercise, L-GykKO mice with high-intensity exercise and LbNOX mice with exercise of both intensities) were not statistically significant (Fig. 4l,p and Extended Data Fig. 6a–d). Muscle ATP levels were similar to those in sedentary mice, except for decreased ATP levels in L-Pck1KO mouse muscles after high-intensity exercise, suggesting their near-complete exhaustion (Fig. 4m,q and Extended Data Fig. 6e–h). Notably, post-exercise accumulation of muscle Pi was larger in the models with decreased exercise capacities than in the corresponding controls (Extended Data Fig. 6i,j). By contrast, there was less muscle Pi accumulation in the models with increased exercise capacities than in the corresponding controls (Fig. 4n,r and Extended Data Fig. 6k,l). Longitudinal training reportedly induces muscle adaptations, such as increases in muscle glycogen content, fatty acid oxidation and mitochondrial electron transport chain-related enzymes^23–25^. On the other hand, except for the upregulated fatty acid transporter *Cd36* in L-Pck1KO mice during low-intensity exercise, enzymes related to fatty acid oxidation and mitochondrial electron transport chain were unchanged (Extended Data Fig. 7a–f). Collectively, these findings indicate that altered gluconeogenesis has profound impacts on muscle ATP production under exercised, but not sedentary, conditions.

In this study, we have shown that hepatic gluconeogenesis utilizing lactate and glycerol has primary roles in muscle ATP production during high-intensity and low-intensity exercise, respectively. Lactate is rapidly produced by muscle glycolysis and thereby acutely reaches its high blood concentrations^26^, matching high-intensity exercise requiring large amounts of glucose production within a short time period. On the other hand, glycerol appears to be suitable for prolonged periods of low-intensity exercise, considering its abundant storage in adipose tissue^27^. Our findings provide insights into the mechanism(s) underlying exercise-induced upregulation of gluconeogenesis: differential increases in gluconeogenic substrate supplies, rather than changes in the expressions of gluconeogenic enzymes, trigger enhanced gluconeogenesis during exercise. In contrast to transcriptional changes with relatively slow and lasting effects, the regulatory mechanism involving the supply of substrates appears to be a system that enables appropriately flexible adjustments to rapidly changing energy demands during exercise. Therefore, the shift of the major gluconeogenic substrate supply, in linkage with distinct hepatic pathways, achieves prompt metabolic adaptations to exercise of different intensities.

Our liver-specific manipulations of gluconeogenesis had minimal effects on enzyme expressions in muscle. From a systemic perspective, cross-talk between muscle and liver through the lactate–glucose carbon recycling system, known as the Cori cycle^26^, modulates high-intensity exercise capacity. In line with our results, liver-specific knockout of both mitochondrial pyruvate carrier 2 and alanine transaminase 2, which impaired PCK1-mediated gluconeogenesis, was reported to decrease exercise capacities with lower blood glucose levels and increased blood lactate levels^28^. Furthermore, upregulated lactate-derived gluconeogenesis in liver-specific prolyl hydroxylase 2 knockout mice reportedly enhanced high-intensity exercise performance^29^. In addition to the classically recognized importance of the Cori cycle in high-intensity exercise^26^, our findings demonstrate that for low-intensity exercise, the adipose–liver–muscle connection mediated by glycerol-derived gluconeogenesis is vital. Viewed collectively, our present results illustrate the important concept that the liver serves as a hub in the inter-organ network, which maintains energy metabolism during exercise through differentially regulated gluconeogenesis utilizing individual redox-dependent substrates (Fig. 4s).

Although fatty acids are known to be a major fuel source for muscle^1,30^, glucose metabolism is considered to greatly affect exercise capacities in our models with hepatic deficiencies of gluconeogenic enzymes. Additionally, considering that mice reportedly rely more heavily on gluconeogenesis for circulating glucose than humans do^30^, the contributions of gluconeogenesis to muscle ATP production might vary among species. Further studies are required to comprehensively elucidate the details of metabolism during exercise and demonstrate its applicability to human exercise.

As evidenced by the isotopic flux analysis, the reciprocally increased exercise capacities were attributable to enhanced gluconeogenesis from glycerol and lactate in L-Pck1KO and L-GykKO mice, respectively. Although a previous study examined the intrahepatic metabolic flux during exercise in congenital liver-specific PCK1 knockout mice with diet-induced obesity^31^, we demonstrate here that redox-dependent gluconeogenesis is differentially enhanced according to exercise intensities, thereby modulating exercise capacities. We have further shown that the hepatic cytosolic [NADH]/[NAD^+^] ratio is crucial for determining capacities for gluconeogenesis from redox-dependent substrates. Interestingly, hepatic PCK1/GYK deletion reduces the cytosolic [NADH]/[NAD^+^] ratio, probably by blockage of the flux of lactate-derived or glycerol-derived gluconeogenesis that consumes cytosolic NAD^+^, which in turn enhances gluconeogenesis through the alternative unblocked redox-dependent pathway. This mechanism may have risk-hedging roles in preventing hypoglycaemia by compensatory augmentation of the alternative gluconeogenic pathway, even if one pathway is impaired. Furthermore, experiments with LbNOX revealed that decreased hepatic cytosolic [NADH]/[NAD^+^] ratios are sufficient to increase exercise capacities through the promotion of gluconeogenesis. In particular, gluconeogenesis was enhanced specifically under conditions with excessive supplies of redox-dependent substrates (that is, during lactate or glycerol tolerance tests and high-intensity or low-intensity exercise) but not under stable conditions (that is, in sedentary states). Thus, this system may function exclusively in response to upregulated redox-dependent substrate supplies, thereby avoiding resting hyperglycaemia. Recent studies have demonstrated that the liver is a bottleneck in metabolic flux during exercise, seemingly because of gluconeogenesis-induced cataplerotic stress; that is, the loss of TCA cycle intermediates^30,32^. Therefore, decreasing hepatic cytosolic [NADH]/[NAD^+^] ratios, which replenish the intermediates by enhancing the influx of redox-dependent substrates, may achieve more efficient metabolic flow of the TCA cycle during exercise.

Methodologies to promote exercise performance have been attracting attention from the viewpoints of both sports and healthier lifestyle interventions^33–35^. Regarding the applicability of our findings to promoting exercise performance, decreasing the hepatic cytosolic [NADH]/[NAD^+^] ratio might be a beneficial approach to enhancing exercise capacities regardless of their intensities. Although 4–8 weeks of endurance training reportedly increased the exercise capacities of C57BL/6 mice by approximately 10–30%^36–39^, hepatic LbNOX expression prolonged exercise durations by 39% and 50% in high-intensity and low-intensity exercise, respectively. Therefore, regulating hepatic cytosolic redox appears to impact exercise capacities, presumably beyond the improvement achieved by long-term training. The liver cytosolic redox state is a promising target for enhancing exercise performance.

## Methods

### Ethics statement

All animal experiments were conducted in accordance with the Tohoku University institutional guidelines. Ethics approval was obtained from the Institutional Animal Care and Use Committee of the Tohoku University Environmental and Safety Committee.

### Generation of *Pck1*-floxed mouse

**Extended Data Fig. 8:**
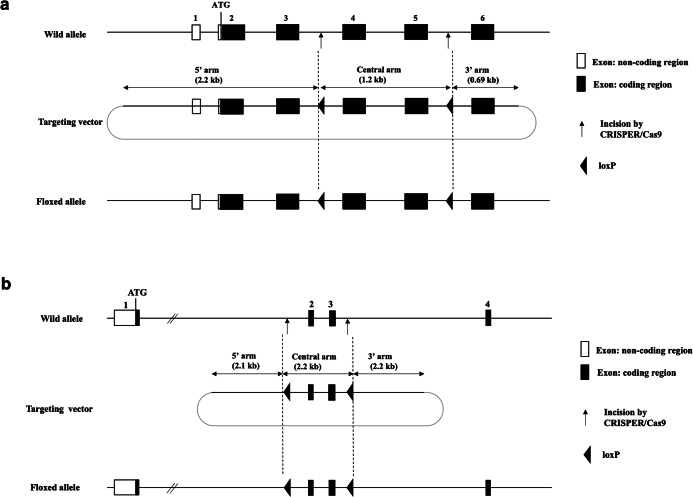
Schematic representations of the vectors used for the generation of *Pck1*- and *Gyk*-floxed mice. (**a**) Schematic representation of the CRISPR/Cas9-mediated gene deletion used for the generation of *Pck1*-floxed mice. Each box represents one exon (numbered). The black boxes represent the *Pck1* coding exons, and the open boxes the non-coding regions. Two mouse genomic sequences (5´- TAG CTG TAG CTA TGG TTC CG -3´and 5´- AAG GTC TGG GAA AGC GCG TA -3´) in introns 3 and 5, respectively, of *Pck1* were selected as guide RNA targets (Arrows). The targeting vector contains six exons of the *Pck1* gene, the fourth and fifth of which are flanked by loxP sites. Homologous recombination between the targeting vector and the *Pck1* locus was confirmed by polymerase chain reaction (PCR). (**b**) Schematic representation of the CRISPR/Cas9-mediated gene deletion used for the generation of *Gyk*-floxed mice. Each box represents one exon (numbered). The black boxes represent the *Gyk* coding exons and the open boxes indicate the non-coding regions. Two mouse genomic sequences (5’- AGC GTA TCG TTC CCA GAC AT -3’) and (5’- TCA ACC AAC CAT GGC AGC AT -3’) in introns 1 and 3, respectively, of *Gyk* were selected as guide RNA targets (Arrows). The targeting vector contains the second and third exons of the *Gyk* gene, flanked by loxP sites. Homologous recombination between the targeting vector and the *Gyk* locus was confirmed by PCR. The details of the methods employed are described in the Materials & Methods section.

To generate *Pck1*-floxed mice for the conditional knockout, two loxP sequences were inserted into the mouse *Pck1* gene locus by homology-dependent repair in mouse zygotes using CRISPR–Cas9 genome editing (Extended Data Fig. 8a). The *Pck1*-floxed mice were generated at the University of Tsukuba (Ibaraki, Japan). All mouse experiments were approved by the University of Tsukuba Animal Experiment Committee. Two mouse genomic sequences (5′-TAGCTGTAGCTATGGTTCCG-3′ and 5′-AAGGTCTGGGAAAGCGCGTA-3′) in introns 3 and 5, respectively, of *Pck1* were selected as guide RNA (gRNA) targets. Each sequence was inserted into a *pX330-mC* plasmid, which carried both the gRNA and Cas9 expression units^40^. The flox donor plasmid DNA, *pflox-Pck1*, carried the genomic region from 907 bp upstream from exon 1 to 220 bp downstream from exon 6 of *Pck1*. Two loxP sequences were inserted at 286 bp upstream from exon 4 and 270 bp downstream from exon 5 in this donor vector. The aforementioned DNA vectors were isolated with a FastGene Plasmid Mini Kit (Nippon Genetics) and filtered with a MILLEX-GV 0.22 μm Filter unit (Merk Millipore) for microinjection. Pregnant mare serum gonadotropin (five units) and human chorionic gonadotropin (five units) were intraperitoneally injected into female C57BL/6J mice with a 48 h interval, followed by mating with male C57BL/6J mice. The zygotes from oviducts in mated females were collected, and mixtures of both *pX330-mC* (circular, 5 ng μl^−1^ each) and *pflox-Pck1* (circular, 10 ng μl^−1^) were microinjected into the zygotes. Subsequently, surviving injected zygotes were transferred into the oviducts of pseudo-pregnant ICR females and newborns were thereby obtained. To confirm the intended flox mutation, the genomic DNA was purified from the tails of G0 mice with PI-200 (Kurabo Industries) according to the manufacturer’s protocol. Genomic PCR was performed with KOD-Fx (TOYOBO). The primers (P_Pck1_-F: 5′-GGCTCGCAGAGAAGTCTTTACAACTGTG-3′ and P_Pck1_-R: 5′-CAGCAAGTGCCTTTACCCATAGACTTGT-3′) were used for confirmation of the correct flox and large deletion mutations. In addition, random integrations of *pX330-mC* and *pflox-Pck1* were checked by PCR with an ampicillin resistance gene detecting primer (P_Amp_-F: 5′-TTGCCGGGAAGCTAGAGTAA-3′ and P_Amp_-R: 5′-TTTGCCTTCCTGTTTTTGCT-3′).

### Generation of *Gyk*-floxed mice

To generate *Gyk*-floxed mice for the conditional knockout, two loxP sequences were inserted into the mouse *Gyk* gene locus by homology-dependent repair in embryonic stem cells using CRISPR–Cas9 genome editing (Extended Data Fig. 8b). Generation of the *Gyk*-floxed mice was performed at Trans Genic (Fukuoka, Japan) with institutional Animal Research Committee approval. To construct the donor vector to insert the two lox sequences, a 2.1 kb mouse genomic fragment containing a part of intron 1 for the 5′ homologous arm, a 2.2 kb fragment containing a part of intron 1, exon 2, intron 2, exon 3 and a part of intron 3 for the floxed region and a 2.2 kb fragment containing a part of intron 3 were amplified by PCR using primers with the loxP sequence from RENKA embryonic stem cell genomic DNA^41^. These fragments were subcloned into plasmids, and the resulting donor vector contained a 2.1 kb 5′ homologous arm, the first loxP site, a 2.2 kb floxed region including exons 2 and 3, the second loxP site and a 2.2 kb 3′ homologous arm. Two mouse genomic sequences (5′-AGCGTATCGTTCCCAGACAT-3′ and 5′-AGCGTATCGTTCCCAGACAT-3′) in introns 1 and 3, respectively, of *Gyk* were selected as gRNA targets. Each gRNA sequence was synthesized as an oligonucleotide DNA, ligated with the U6 promoter and inserted into the plasmid containing the CAG promoter-driven hCas9 coding sequence to construct the expression vector. To validate the efficiency of the gene-targeted double-strand break using the designed sgRNA and hCas9, reconstitution of green fluorescence by homology-dependent repair of EGFP was examined^42^. The donor vector, two expression vectors and a plasmid containing the PGK-driven puromycin-resistant gene for transfectant selection were introduced into RENKA embryonic stem cells (C57BL/6N) by electroporation. After selection using puromycin, the resistant clones were isolated, and their DNA samples were screened for loxP insertion by PCR using the following primer sets: P_Gyk_5-F: 5′-CCCCCGCGGCCGCCTGAGGTTCTCATAGTTACACATCC-3′ and P_Gyk_5-R: 5′-AGGGGCCTATGGGCCCATAACTTCG-3′ to screen for 5′ loxP insertion, and P_Gyk_3-F: 5′-CCTATGCTGCCGGATCCATAACTTC-3′ and P_Gyk_3-R: 5′-CTCGAGGTCGACTGTGTCTGAGTCTAGTCTGGTCTCC-3′ to screen for 3′ loxP insertion. PCR-positive embryonic stem clones were expanded, and isolated DNA samples were further analysed by PCR amplification followed by restriction enzyme digestion to examine whether the two loxP sites are on the same allele. The primer set used in this PCR was P_Gyk_5-F and P_Gyk_3-R to amplify from intron 1 to intron 3, including the loxP insertion sequence. Amplified fragments were digested with PspOM1, the recognition sequence of which was introduced with the 5′ loxP sequence, and BamHI, the recognition sequence of which was introduced with the 3′ loxP sequence. Clones that showed a band at the double-digested size were considered to have the two loxP sites in a single allele (floxed allele). The insertion of loxP sites at the expected region was also confirmed by direct sequencing. Recombinant embryonic stem cell clones with the floxed region were also analysed by genomic Southern hybridization probed with the genomic sequence of the floxed region to confirm that these clones did not contain random integration of the donor vector. Recombinant embryonic stem cell clones with the floxed allele were aggregated with ICR eight-cell stage embryos to generate chimeric mice. Germline-transmitting F1 heterozygous mice were obtained by crossing chimeric mice with a high contribution of the RENKA embryonic stem cells and C57BL/6N mice. The targeted allele was identified by PCR using primer sets P_Gyk_5-F–P_Gyk_5-R and P_Gyk_3-F*–*P_Gyk_3-R, genomic Southern hybridization and direct sequencing in F1 mice.

### Animals

All mice were group-housed in a plastic cage with a 12 h light, 12 h dark cycle and received a normal chow diet (MF Diet, cat. no. MF, Oriental Yeast) ad libitum. C57BL6/J or C57BL6/N mice were obtained from CLEA Japan. To obtain tamoxifen-inducible liver-specific knockout mice, we used transgenic mice expressing a Cre recombinase transgene fused to mutated estrogen receptor ligand-binding domains under the control of the serum albumin promoter (SA-CreER^T2^ mice)^43,44^. To generate tamoxifen-inducible liver-specific PCK1 knockout mice (L-Pck1KO mice), we crossed SA-CreER^T2^ mice and *Pck1-*floxed mice. SA-CreER^T2^ −/−; *Pck1*-floxed mice (control mice) and SA-CreER^T2^ +/−; *Pck1*-floxed mice (L-Pck1KO mice) were used for experiments. To generate tamoxifen-inducible liver-specific GYK knockout mice (L-GykKO mice), we crossed SA-CreER^T2^ mice and *Gyk-*floxed mice. SA-CreER^T2^ −/−; *Gyk*-floxed mice (control mice) and SA-CreER^T2^ +/−; *Gyk*-floxed mice (L-GykKO mice) were used for experiments. To induce a knockout, mice were intraperitoneally administered 1 mg of tamoxifen for three consecutive days, 1 week before the experiments. All experiments were conducted using male mice at 16–18 weeks of age unless otherwise indicated. Experiments were performed under 24 h fasted conditions, at which point liver glycogen is almost entirely depleted except as otherwise provided. For measurements of liver metabolites, mice were anaesthetized with 2.5–3% isoflurane, and the liver was rapidly frozen by freeze-clamp within a few seconds after removal to avoid degradation of metabolites^12,45,46^. Other organs were frozen in liquid nitrogen immediately after removal. For measurements of muscle metabolites during the exercise experiments, mice were killed by cervical dislocation, and the sampled gastrocnemius was rapidly frozen in liquid nitrogen.

### Exercise experiments

A motorized treadmill (MK680, Muromachi Kikai) was used for all exercise experiments. Before the exercise experiments, mice were adapted to running on a treadmill at the indicated speed for six and three consecutive days before high-intensity and low-intensity exercise, respectively. The detailed protocols are shown in Supplementary Table 1. All pre-adaptations were conducted during the dark cycle. The exercise experiments started at 09:00 h (1 h after the onset of the dark cycle). In the high-intensity exercise protocol, mice started to run at 10 m min^−1^, and the speed was increased incrementally by 1 m min^−1^ every 1 min up to 25 m min^−1^; the mice then ran until exhaustion or for 20 min. With the low-intensity exercise protocol, mice ran at 13 m min^−1^ until exhaustion or for 60 min. The sedentary group of mice were placed on the treadmill machine without running (at 0 m min^−1^) for the same period of time as the exercised mice. Exhaustion was defined as the inability to continue running with the mouse staying in the electrical shock grid for five consecutive seconds.

### Isotopic metabolic flux analysis during exercise

Mice were treated with tamoxifen and adapted to treadmill running as above for 2 weeks before the exercise experiment. Catheters (C10PU-MCA1301, Instech Laboratories) were implanted in the left common carotid artery for blood sampling and the right jugular vein for isotope infusion 1 week before the experiment, as previously reported^47^. The free ends of catheters were plugged by PinPorts (PNP3F22, Instech Laboratories) after tunnelling under the skin to the back of the neck. Mice with a weight loss of over 10% on the experimental day compared with the pre-surgery weight were excluded. Food was removed 24 h before exercise. After placing the mice on the treadmill, blood was collected (*t*_0_). Then, primed constant infusions of stable isotopes were started 210 min before exercise as previously reported^31^. For the low-intensity exercise experiment on L-Pck1KO and their controls, [6,6-^2^H_2_]glucose (priming with 400 μmol kg^−1^ prime followed by continuous infusion of 40 μmol kg^−1^ min^−1^) (661414, Sigma-Aldrich) and [^13^C_3_]glycerol (priming with 40 μmol kg^−1^ followed by continuous infusion of 4 μmol kg^−1^ min^−1^) (CLM-1510-0, Cambridge Isotope Laboratories) were infused. For the high-intensity exercise experiment on L-GykKO and their controls, [6,6-^2^H_2_]glucose (priming with 400 μmol kg^−1^ for the prime infusion followed by continuous infusion of 40 μmol kg^−1^ min^−1^) and [^13^C_3_]lactate (priming with 150 μmol kg^−1^ followed by continuous infusion of 15 μmol kg^−1^ min^−1^) (746258, Sigma-Aldrich) were infused. Under the sedentary conditions, blood samples were collected at two different timepoints of approximately 20 min and 10 min before exercise and then recorded as *t*_1_ and *t*_2_, respectively. Blood samples were also collected during exercise. The timepoints were approximately 20 min (*t*_3_) and 25 min (*t*_4_) after starting high-intensity exercise or about 60 min (*t*_3_) and 70 min (*t*_4_) after starting low-intensity exercise. Plasma samples were collected after centrifugations of blood samples and stored at −80°C.

### Sample analysis and calculations for metabolic flux

The concentration (*C*) and isotopic enrichment (IE) of glucose at each timepoint were measured using ion chromatography coupled with high-resolution mass spectrometry. An Orbitrap mass spectrometer (Q-Exactive Focus) and a high-performance ion chromatography system (ICS-5000+) enabled sensitive and selective detection based on anion separation and accurate mass measurement^48^. IE at each timepoint was corrected by subtracting naturally occurring isotopes (IE at *t*_0_ = IE_0_). The glucose rate of appearance (Ra) was calculated using Steel’s non-steady-state equation modified for stable-isotope experiments^8,49^,

$${\rm{Glucose}}\;{\rm{Ra}}=\frac{{F}-{V}(({C}_{{\rm{m}}}+{{C}}_{{\rm{n}}})/2)(({{\rm{IE}}}_{{\rm{n}}}-{{\rm{IE}}}_{{\rm{m}}})/({{t}}_{{\rm{n}}}-{{t}}_{{\rm{m}}}))}{({{\rm{IE}}}_{{\rm{m}}}+{{\rm{IE}}}_{{\rm{n}}})/2}$$

where *F* is the infusion rate of [6,6-^2^H_2_]glucose (40 μmol kg^−1^ min^−1^) and *V* is the volume of distribution, which was assumed to be 40 ml kg^−1^. Glucose rate of appearance was calculated using IE for [6,6-^2^H_2_]glucose. Values at *t*_1_ and *t*_2_ (m = 1 and n = 2) were used to obtain the metabolic flux rates under sedentary conditions, while metabolic flux rates during exercise were calculated using values at *t*_3_ and *t*_4_ (m = 3 and n = 4). The contents of ^13^C-labelled glucose were calculated as the average ratio to [^12^C_6_]glucose at *t*_3_ and *t*_4_.

### RNA purification and quantitative PCR with reverse transcription

Using the RNAeasy mini kit (Qiagen), total RNA was extracted from mouse liver or gastrocnemius, which had been stored in RNAprotect Tissue Reagent (Qiagen). Complementary DNA, synthesized from 1 μg of total RNA using a ReverTra Ace qPCR RT Master Mix (TOYOBO), was evaluated with a CFX96 Touch Real-Time PCR Detection System (Bio-Rad). mRNA expression levels were normalized to the levels of *hydroxymethylbilane synthase* (*Hmbs*) in the liver and *glyceraldehyde-3-phosphate dehydrogenase* (*Gapdh*) in gastrocnemius. The sequences of the primers are listed in Supplementary Table 2.

### Western blotting analysis

Mouse tissues were homogenized in lysis buffer (100 mM Tris pH 8.5, 250 mM NaCl, 1 mM EDTA, 1% NP-40) containing protease inhibitors (35 mg ml^−1^ phenylmethylsulfonyl fluoride, 10 mg ml^−1^ aprotinin and 10 mg ml^−1^ leupeptin). After centrifugation at 20,400*g* for 5 min, the protein concentrations of supernatants were determined using a Pierce BCA Protein Assay Kit (Thermo Fisher Scientific). Equal amounts of proteins with Laemmli SDS sample buffer were boiled for 5 min. Next, 30 μg of tissue protein extracts were loaded onto an SDS–PAGE column and then transferred to a nitrocellulose membrane. After blocking with Tris-buffered saline containing 2–5% skim milk, membranes were incubated with antibodies to GAPDH (5174S, Cell Signaling Technology), GYK (ab126599, Abcam), PCK1 (16754-1-AP, Proteintech), pyruvate carboxylase (16588-1-AP, Proteintech), fructose-1,6-bisphosphatase 1 (ab109732, Abcam) or FLAG (2368S, Cell Signaling Technology). After washing, the membranes were incubated with anti-rabbit horseradish peroxidase-linked IgG secondary antibody (NA9340V, GE HealthCare Japan). Chemiluminescence signal enhancement caused by the Pierce ECL Plus Western Blotting Substrate (Thermo Fisher Scientific) was detected with a ChemiDoc Touch Imaging System (Bio-Rad). Quantitative data were obtained using ImageJ software.

### Tissue metabolite and blood analyses

Metabolites in the liver and gastrocnemius muscle extracts were measured after deproteinization with perchloric acid (26503-75, Nacalai Tesque). Blood samples were obtained from the tail vein. Liver and muscle glycogen levels were measured after amyloglucosidase digestion as previously described^50,51^. Commercial kits were used for measuring lactate (K607, BioVision), pyruvate (K609, BioVision), alanine (MET-5093, Cell Biolabs), free fatty acids (279-75401, FUJIFILM Wako), ketone bodies (ab272541, Abcam), NAD(H) (N509, Dojindo), ATP (ab83355, Abcam) and Pi (ab65622, Abcam) according to the relevant instructions for each.

### Tolerance tests of gluconeogenic substrates

Mice were intraperitoneally administered 1 g kg^−1^ body weight of gluconeogenic substrates diluted in PBS. The substrates were glycerol (075-00611, FUJIFILM Wako), DHA (NA-0183, Combi-Blocks), lactate (L7022, Sigma-Aldrich), pyruvate (P8574, Sigma-Aldrich) and alanine (010-01042, FUJIFILM Wako). Glucose levels in blood from the tail vein were measured using a Glutestmint Kit (Sanwa Kagaku Kenkyusho) before and 15, 30, 60 and 120 min after injections of the substrates.

### Primary hepatocyte experiments

Primary hepatocytes were isolated by perfusion and digestion using Liberase (Roche) as previously reported^52^. Dead cells were removed using Percoll (17-0891-01, GE HealthCare Japan). Isolated hepatocytes were seeded at 10^5^ cells per well into 12-well collagen-coated dishes in DMEM (11995065, Thermo Fisher Scientific) supplemented with 10% FBS, 1% penicillin–streptomycin, 10 mM HEPES, 1 nM dexamethasone and 0.5 U ml^−1^ insulin. After overnight incubation at 37°C in a 5% CO_2_ chamber, cells were washed three times with PBS, followed by a 2 h incubation in no-glucose DMEM (1443001) supplemented with 10 mM HEPES. After the cells had been washed three times with PBS, they were incubated for 3 h in 10 mM HEPES no-glucose DMEM containing 10 mM glycerol or lactate with or without 10 mM ethanol. Glucose levels in media were measured with normalization to total protein concentrations from hepatocytes lysed in 1% SDS and expressed as a percentage of the glucose amount produced in the absence of a substrate.

### Adenovirus experiments

FLAG-tagged LbNOX was generated with a ViraPower Adenoviral Expression Kit (K493000, Thermo Fisher Scientific) using a subcloned gene from pUC57-LbNOX. pUC57-LbNOX was a gift from V. Mootha (Addgene, plasmid 75285; http://n2t.net/addgene:75285;RRID:Addgene_75285)^17^. LacZ adenovirus was used as a control. Adenovirus (1 × 10^9^ plaque-forming units) was intravenously injected into 16-week-old wild-type C57BL/6J mice, C57BL/6N mice or knockout mice and the corresponding controls 1 week after tamoxifen treatment.

### Statistics and reproducibility

No statistical method was used to predetermine sample size. However, the sample sizes used in this study are consistent with those in previous publications^9,29,31^ and were considered sufficient based on our prior experience with similar experimental designs^44,53^. Mice with abnormal body weights were excluded before the experiments. In the isotopic flux analysis (Fig. 2i–m), mice with > 10% weight loss on the day of the experiment (compared to pre-surgery weight) or with technical failures (for example, catheter withdrawal) were excluded. In Extended Data Fig. 2m, one epididymal white adipose tissue weight measurement was missing in the L-GykKO group and was therefore excluded. No other data points were excluded. Animals were randomly assigned to experimental groups after matching for body weight. Data collection and analysis were not performed blind to the conditions of the experiments.

Data distribution was assumed to be normal, but this was not formally tested. All statistical tests were two-sided. For comparisons between two groups, two-tailed unpaired *t*-tests were used. For comparisons involving more than two groups without a time component (that is, bar graphs), one-way ANOVA followed by Tukey’s post hoc analysis was performed. For time-course experiments (that is, line graphs), repeated measures two-way ANOVA was used when all groups had the same number of samples (balanced design), and a mixed-effects model based on restricted maximum likelihood was used when group sizes differed (unbalanced design). In both cases, Holm–Šídák post hoc analyses were applied for multiple comparisons. For Holm–Šídák analyses, comparisons were restricted to predefined relevant comparisons; specifically, within-timepoint comparisons between genotypes or treatment groups (for example, control vs KO or Ad-LacZ vs Ad-LbNOX at each timepoint), and within-group comparisons across time (for example, pre vs post within control or KO). Cross-group timepoint comparisons (for example, pre–control vs post–KO) were not conducted. In cases for which no significant main effects or interactions were detected by ANOVA or mixed-effects models, post hoc analysis was not performed. Data are presented as means ± s.e.m. Each plot on the bar graph shows raw data. *P* values are denoted in figures as not significant (ns), *P < 0.05 or **P < 0.01. Exact *P* values and sample sizes (*n*) for each group are provided in the figure legends. Prism 10 (GraphPad Software) was used for all statistical analyses.

### Reporting summary

Further information on research design is available in the Nature Portfolio Reporting Summary linked to this article.

## Supplementary information

Supplementary Information Supplementary Tables 1 and 2.

Reporting Summary

## Source data

Source Data Fig. 1 Statistical source data.

Source Data Fig. 2 Statistical source data.

Source Data Fig. 3 Statistical source data.

Source Data Fig. 4 Statistical source data.

Source Data Extended Data Fig. 1 Statistical source data.

Source Data Extended Data Fig. 2 Statistical source data.

Source Data Extended Data Fig. 3 Statistical source data.

Source Data Extended Data Fig. 4 Statistical source data.

Source Data Extended Data Fig. 5 Statistical source data.

Source Data Extended Data Fig. 6 Statistical source data.

Source Data Extended Data Fig. 7 Statistical source data.

Source Data Fig. 1 Uncropped western blots.

Source Data Extended Data Fig. 2 Uncropped western blots.

Source Data Extended Data Fig. 4 Uncropped western blots.

Source Data Extended Data Fig. 5 Uncropped western blots.

## Data Availability

No datasets were generated or analysed during the current study. Source data are provided with this paper.
