# Supplementary material for: Redox-dependent liver gluconeogenesis impacts different intensity exercise in mice
Source: Nat Metab. 2025 Sep 18;7(10):1991–2003. doi: 10.1038/s42255-025-01373-z (PMC12552127; doi:10.1038/s42255-025-01373-z)
Supplement: Supplementary file 1 — Supplementary Tables 1 and 2. [file 42255_2025_1373_MOESM1_ESM.pdf]

nature metabolism

Supplementary information

https://doi.org/10.1038/s42255-025-01373-z

Redox-dependent liver gluconeogenesis impacts different intensity exercise in mice

In the format provided by the authors and unedited

Supplementary Table 1 The protocols for exercise experiments

<High-intensity exercise protocol>

Day -7

Time(min)	Speed (m/min)
0	8
5	9
7	10
10	End

Day -6

Time(min)	Speed(m/min)
0	10
5	11
10	12
15	End

Day -5

Time(min)	Speed (m/min)
0	10
1	11
2	12
3	13
4	14
5	15
15	End

Day -4

Time(min)	Speed (m/min)
0	10
1	11
2	12
3	13
4	14
5	15
6	16
7	17
8	18
9	19
10	20
15	End

Day -3

Time(min)	Speed (m/min)
0	10
1	11
2	12
3	13
4	14
5	15
6	16
7	17
8	18
9	19
10	20
15	End

Day -2

Time(min)	Speed (m/min)
0	10
1	11
2	12
3	13
4	14
5	15
6	16
7	17
8	18
9	19
10	20
11	21
12	22
13	23
14	24
15	End

Day 0 (test)

Time(min)	Speed (m/min)
0	10
1	11
2	12
3	13
4	14
5	15
6	16
7	17
8	18
9	19
10	20
11	21
12	22
13	23
14	24
15	25
20 or exhaustion	End

<Low-intensity exercise protocol>

Day -4

Time (min)	Speed (m/min)
0	8
5	9
7	10
10	End

Day -3, -2

Time (min)	Speed(m/min)
0	10
5	11
10	12
15	End

Day 0 (test)

Time (min)	Speed(m/min)
0	13
60 or exhaution	End

Supplementary Table 2 The list of primers for qPCR

Gene		Sequence (5'-3')
Hmbs	Forward	ATGAGGGTGATTCGAGTGGG
	Reverse	TTGTCTCCCGTGGTGGACATA
Gyk	Forward	CAACTCAGTCTCCAGAAAGTGGTAT
	Reverse	CTTAGAGGATCATGCAGCCAGT
Pck1	Forward	TTGCCTGGATGAAGTTTGAT
	Reverse	GGCATTTGGATTTGTCTTCACT
Pck2	Forward	GTCATGGGGTGTTTGTAGGTAG
	Reverse	ACCAGTGTTCCAGGTAGCGT
Fbp1	Forward	CTATGGTATCGCTGGCTCAAC
	Reverse	ATCAAGGGGATCGAAACAGAC
Pcx	Forward	ACCTAGGTGCTTGGCTGGTA
	Reverse	CATTTGGGGAGGCAACAG
Gapdh	Forward	TGAAGGTCGGTGTGAACG
	Reverse	CCATTCTCGGCCTTGACT
Slc27a1	Forward	CTACCACTCTGCAGGGAACA
	Reverse	GGTAGCGGCAGATTTCACCT
Cd36	Forward	TGGCCAAGCTATTGCGACAT
	Reverse	ACACAGCGTAGATAGACCTGC
Cpt1b	Forward	TGGTGGGCAACTAACTATGTGA
	Reverse	TCCAGTTTGCGGCGATACAT
Ndufa2	Forward	GTTGCGTGAGATTCGCGTTC
	Reverse	GCACGATGAAATCCCTCACAC
Uqcrc2	Forward	CCGGGTCCTTCTCGAGATTTTA
	Reverse	CCGATTCTTGACAGAGGAGCA
Cycs	Forward	AGGCAAGCATAAGACTGGACC
	Reverse	CTCTCCCCAGGTGATGCCTTT
Cox4i	Forward	CCTTGGACGGCGGAATG
	Reverse	AGACAGCATCGTGACATGGG
Atp5b	Forward	TGAGAGAGGTCCTATCAAAACCA
	Reverse	CACCAGAATCTCCTGCTCAAC
