# Supplementary material for: Redox-dependent liver gluconeogenesis impacts different intensity exercise in mice
Source: Nat Metab. 2025 Sep 18;7(10):1991–2003. doi: 10.1038/s42255-025-01373-z (PMC12552127; doi:10.1038/s42255-025-01373-z)
Supplement: Supplementary file 2 — Reporting Summary [file 42255_2025_1373_MOESM2_ESM.pdf]

nature portfolio

Corresponding author(s): Katagiri Hideki, Keizo Kaneko

Last updated by author(s): Jul 22, 2025

# Reporting Summary

Nature Portfolio wishes to improve the reproducibility of the work that we publish. This form provides structure for consistency and transparency in reporting. For further information on Nature Portfolio policies, see our Editorial Policies and the Editorial Policy Checklist.

## Statistics

For all statistical analyses, confirm that the following items are present in the figure legend, table legend, main text, or Methods section.

| n/a | Confirmed | |
|---|---|---|
| ☐ | ☒ | The exact sample size (*n*) for each experimental group/condition, given as a discrete number and unit of measurement |
| ☐ | ☒ | A statement on whether measurements were taken from distinct samples or whether the same sample was measured repeatedly |
| ☐ | ☒ | The statistical test(s) used AND whether they are one- or two-sided<br>*Only common tests should be described solely by name; describe more complex techniques in the Methods section.* |
| ☒ | ☐ | A description of all covariates tested |
| ☐ | ☒ | A description of any assumptions or corrections, such as tests of normality and adjustment for multiple comparisons |
| ☐ | ☒ | A full description of the statistical parameters including central tendency (e.g. means) or other basic estimates (e.g. regression coefficient) AND variation (e.g. standard deviation) or associated estimates of uncertainty (e.g. confidence intervals) |
| ☐ | ☒ | For null hypothesis testing, the test statistic (e.g. *F*, *t*, *r*) with confidence intervals, effect sizes, degrees of freedom and *P* value noted<br>*Give P values as exact values whenever suitable.* |
| ☒ | ☐ | For Bayesian analysis, information on the choice of priors and Markov chain Monte Carlo settings |
| ☒ | ☐ | For hierarchical and complex designs, identification of the appropriate level for tests and full reporting of outcomes |
| ☒ | ☐ | Estimates of effect sizes (e.g. Cohen's *d*, Pearson's *r*), indicating how they were calculated |

*Our web collection on statistics for biologists contains articles on many of the points above.*

## Software and code

Policy information about availability of computer code

| | |
|---|---|
| Data collection | Microsoft Excel for Mac. |
| Data analysis | Graphpad Prism for macOS (v10) |

For manuscripts utilizing custom algorithms or software that are central to the research but not yet described in published literature, software must be made available to editors and reviewers. We strongly encourage code deposition in a community repository (e.g. GitHub). See the Nature Portfolio guidelines for submitting code & software for further information.

## Data

Policy information about availability of data

All manuscripts must include a data availability statement. This statement should provide the following information, where applicable:

- Accession codes, unique identifiers, or web links for publicly available datasets
- A description of any restrictions on data availability
- For clinical datasets or third party data, please ensure that the statement adheres to our policy

All data supporting the findings of this study are available within the manuscript. Raw data are presented in bar graps, and source data are provided with this paper. No external datasets were generated or analyzed during the current study.

## Research involving human participants, their data, or biological material

Policy information about studies with human participants or human data. See also policy information about sex, gender (identity/presentation), and sexual orientation and race, ethnicity and racism.

| | |
|---|---|
| Reporting on sex and gender | not applicable |
| Reporting on race, ethnicity, or other socially relevant groupings | not applicable |
| Population characteristics | not applicable |
| Recruitment | not applicable |
| Ethics oversight | not applicable |

Note that full information on the approval of the study protocol must also be provided in the manuscript.

# Field-specific reporting

Please select the one below that is the best fit for your research. If you are not sure, read the appropriate sections before making your selection.

☒ Life sciences ☐ Behavioural & social sciences ☐ Ecological, evolutionary & environmental sciences

For a reference copy of the document with all sections, see nature.com/documents/nr-reporting-summary-flat.pdf

# Life sciences study design

All studies must disclose on these points even when the disclosure is negative.

| | |
|---|---|
| Sample size | No statistical method was used to predetermine sample size. Sample sizes were based on our prior experience with similar experiments and are consistent with those reported in previous publications. |
| Data exclusions | Mice with abnormal body weights were excluded prior to the experiments. In the isotopic flux analysis (Figures 2i–m), mice with >10% weight loss compared to pre-surgery or with technical failures (e.g., catheter withdrawal) were excluded. In Extended Data Figure 2m, one epididymal WAT weight value was missing (not recorded) in the L-GykKO group and was excluded. No other data were excluded. |
| Replication | Key experiments with exercise capacities and changes of plasma metabolite concentrations (FIgures 1f-m,2a-h, 4c-l), and experiments related to gluconeogenic responses (Figures 3a-j, 4a,b) were replicated in independent experiments. In main figures, experiments with isotopic flux (Figures 2i-m) were not replicated at this stage, due to its extended duration of the exerperiments. |
| Randomization | Mice of the same sex and age were used in each experiment. Animals were randomly assigned to experimental groups after matching for body weight. |
| Blinding | Formal blinding was not performed due to technical constraints requiring knowledge of group allocation. For example, treadmill running involved simultaneous exercise of multiple mice, making it necessary to identify individual animals during the experiment. |

# Reporting for specific materials, systems and methods

We require information from authors about some types of materials, experimental systems and methods used in many studies. Here, indicate whether each material, system or method listed is relevant to your study. If you are not sure if a list item applies to your research, read the appropriate section before selecting a response.

### Materials & experimental systems

| n/a | Involved in the study | |
|---|---|---|
| ☐ | ☒ | Antibodies |
| ☒ | ☐ | Eukaryotic cell lines |
| ☒ | ☐ | Palaeontology and archaeology |
| ☐ | ☒ | Animals and other organisms |
| ☒ | ☐ | Clinical data |
| ☒ | ☐ | Dual use research of concern |
| ☒ | ☐ | Plants |

### Methods

| n/a | Involved in the study | |
|---|---|---|
| ☒ | ☐ | ChIP-seq |
| ☒ | ☐ | Flow cytometry |
| ☒ | ☐ | MRI-based neuroimaging |

## Antibodies

Antibodies used

All antibodies were used for western blotting.

Primary antibodies:
GAPDH (#5174S, Cell Signaling Technology; 1:2,000)
GYK (ab126599, Abcam; 1:2,000)
PCK1 (16754-1-AP, Proteintech; 1:2,000)
PC (16588-1-AP, Proteintech; 1:2,000)
FBP1 (ab109732, Abcam; 1:2,000)
FLAG (#2368, Cell Signaling Technology; 1:2,000)

Secondary antibody:
anti-rabbit horseradish peroxidase-linked IgG secondary antibody (NA9340V, GE Health Care Japan; 1:10,000)

Validation

All antibodies were commercially obtained. See following links for validation.

Primary antibodies:
GAPDH (#5174S, Cell Signaling Technology) https://www.cellsignal.com/products/primary-antibodies/gapdh-d16h11-xp-rabbit-mab/5174?site-search-type=Products&N=4294956287&Ntt=5174s&fromPage=plp&_requestid=688970
GYK (ab126599, Abcam) https://www.abcam.com/products/primary-antibodies/glycerol-kinase-antibody-epr6567-ab126599.html
PCK1 (16754-1-AP, Proteintech) https://www.ptglab.com/products/PCK1-Antibody-16754-1-AP.htm
PC (16588-1-AP, Proteintech) https://www.ptglab.com/products/PC-Antibody-16588-1-AP.htm
FBP1 (ab109732, Abcam) https://www.abcam.co.jp/primary-antibodies/fbp1-antibody-epr4620-ab109732.html
FLAG (#2368, Cell Signaling Technology) https://www.cellsignal.com/products/primary-antibodies/dykddddk-tag-antibody-binds-to-same-epitope-as-sigma-aldrich-anti-flag-m2-antibody/2368?srsltid=AfmBOopjpH36gUUscbxwxE_2fdHnPxsObGVFfesD7qc2m3VBM6rLQa5I

Secondary antibody:
anti-rabbit horseradish peroxidase-linked IgG secondary antibody (NA9340V, GE Health Care Japan) https://gels.yilimart.com/Assets/Images/doc/file/NA934-1ML_INSTRUCTION_09.PDF

## Animals and other research organisms

Policy information about studies involving animals; ARRIVE guidelines recommended for reporting animal research, and Sex and Gender in Research

Laboratory animals

Mice were housed in groups of 2-5 animals per cage under a 12-hour light/dark cycle at an ambient temperature of 23-27°C and 40-60% humidity. All experiments were conducted at 16-18 weeks of age. Male C57BL/6J and C57BL/6N mice at 8-12 weeks of age were purchased from CLEA Japan. L-GykKO and L-Pck1KO mice were obtained by crossing SA-CreERT2 mice with Gyk- and Pck1-floxed mice, respecitively. All mice were of C57BL/6 background. The detailed methods for generating Gyk- and Pck1-floxed mice are within the manuscript.

Wild animals

No wild animals were used in this study.

Reporting on sex

Male and female mice were used in this study as indicated.

Field-collected samples

No field-collected samples were used in this study.

Ethics oversight

All animal experiments were conducted in accordance with the Tohoku University institutional guidelines. Ethics approval was obtained from the Institutional Animal Care and Use Committee of the Tohoku University Environmental & Safety Committee.
Generation of Gyk-floxed mice was performed at Trans Genic Inc. (Fukuoka, Japan) with institutional Animal Research Committee approval.
Generation of Pck1-floxed mice was performed at the University of Tsukuba (Ibaraki, Japan) with the approval by the University of Tsukuba Animal Experiment Committee.

Note that full information on the approval of the study protocol must also be provided in the manuscript.
