# Supplementary figures and images for: Redox-dependent liver gluconeogenesis impacts different intensity exercise in mice
Source: Nat Metab. 2025 Sep 18;7(10):1991–2003. doi: 10.1038/s42255-025-01373-z (PMC12552127; doi:10.1038/s42255-025-01373-z)

Figure 1d

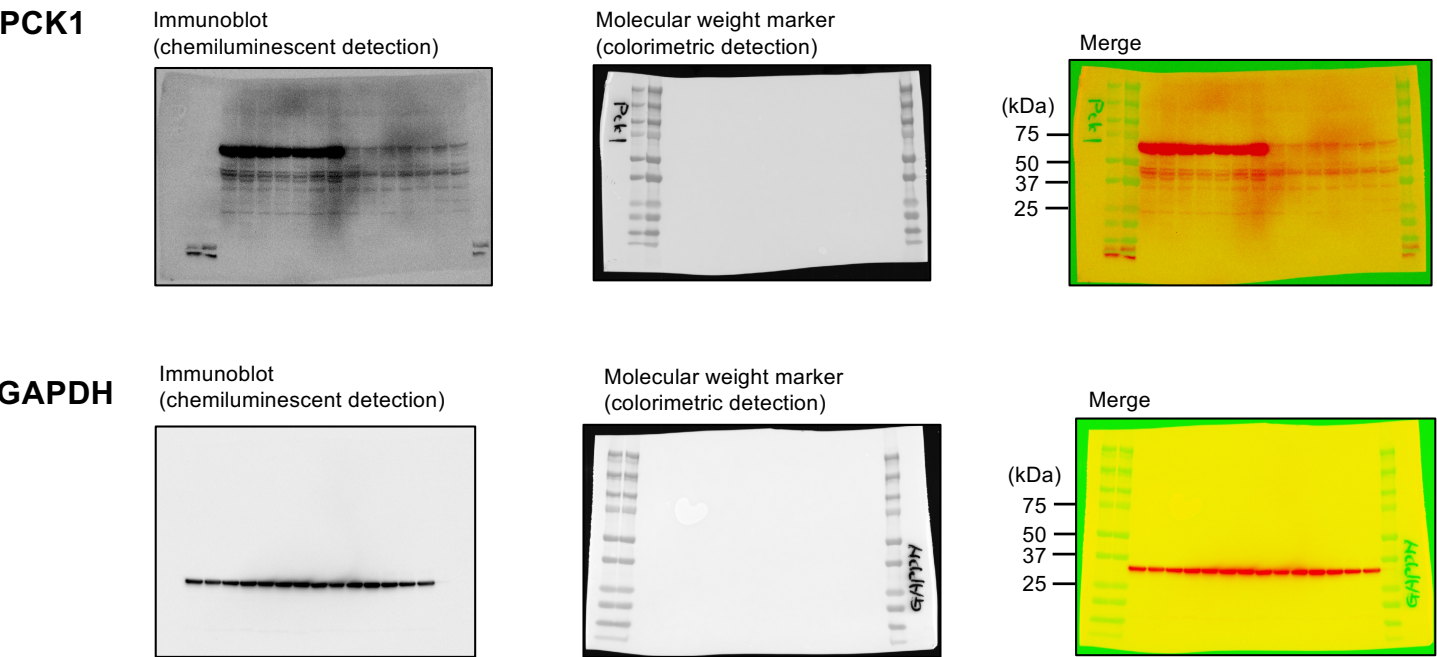

Figure 1e

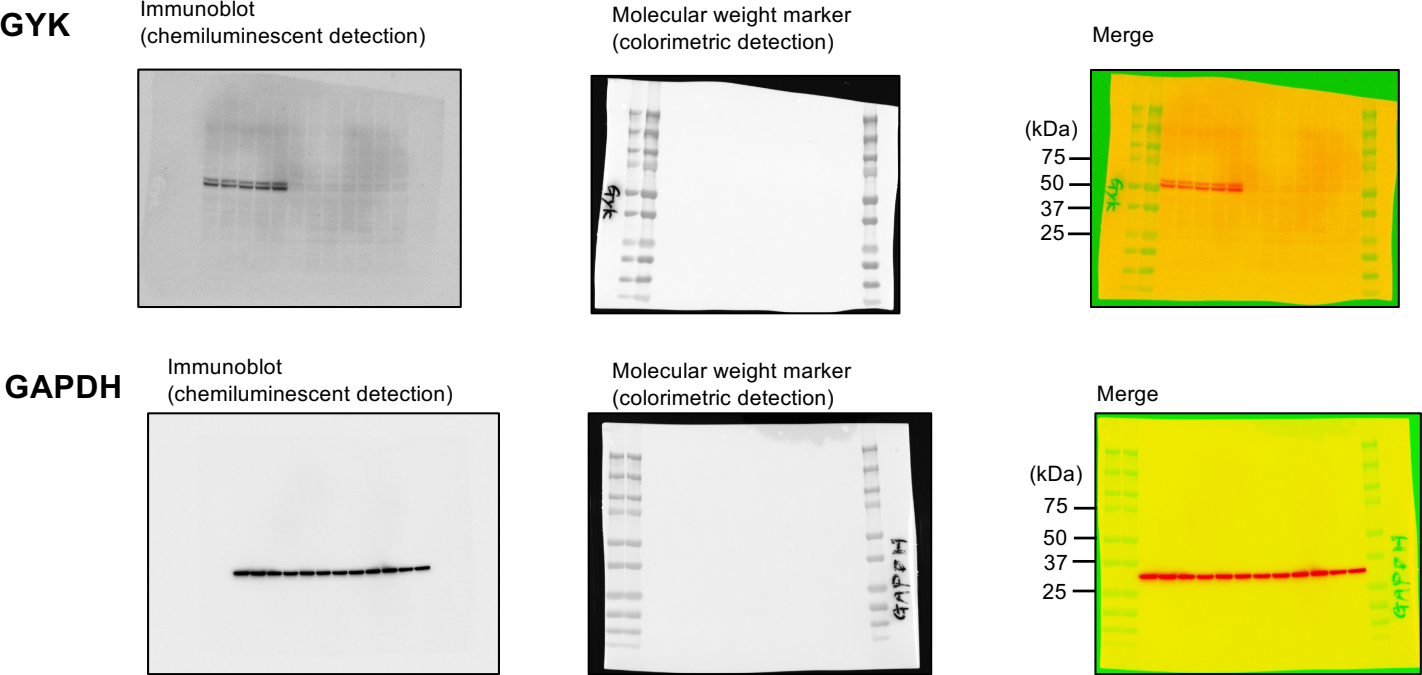

Supplement: Supplementary file 14 — Uncropped western blots. [file 42255_2025_1373_MOESM14_ESM.pdf]

Extended Data Fig. 4a

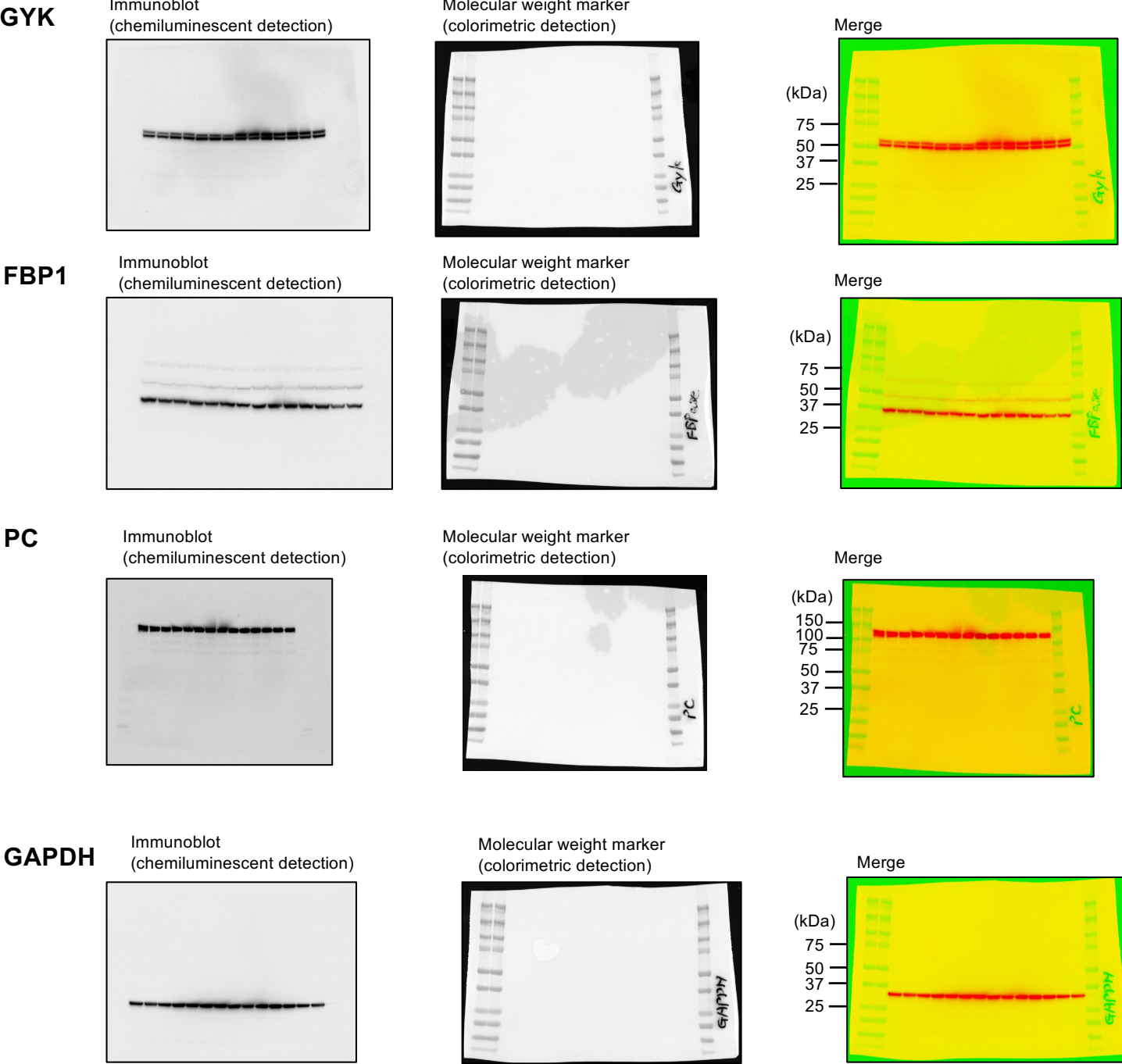

Extended Data Fig. 4b

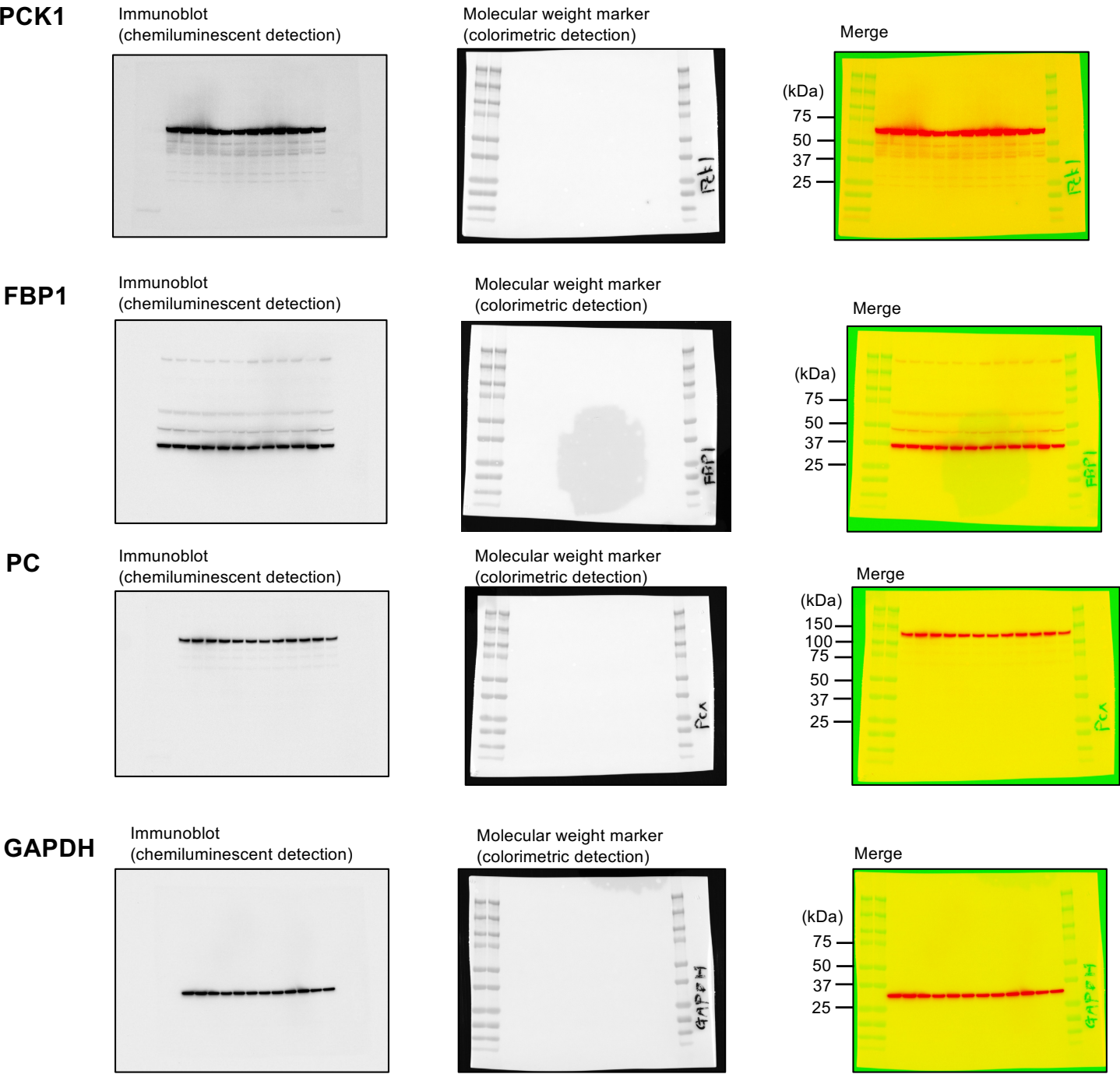

Supplement: Supplementary file 16 — Uncropped western blots. [file 42255_2025_1373_MOESM16_ESM.pdf]

Extended Data Fig. 5a

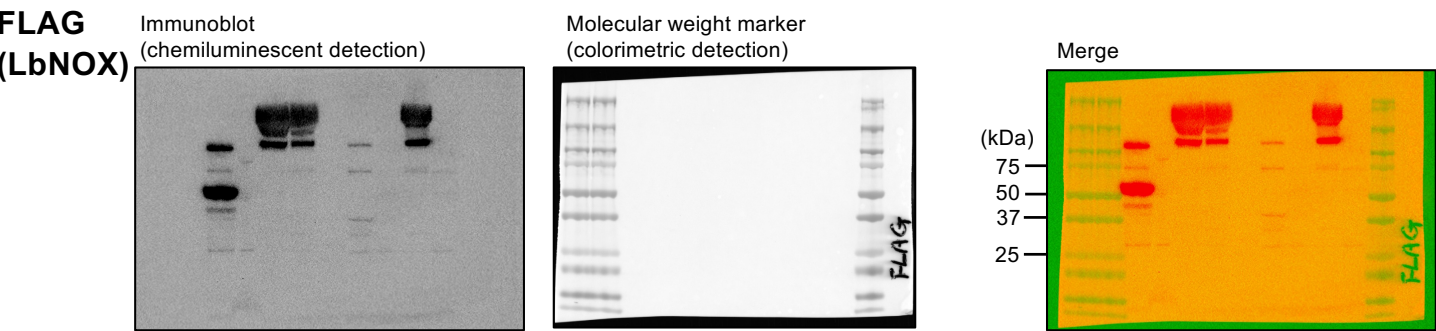

Extended Data Fig. 5h

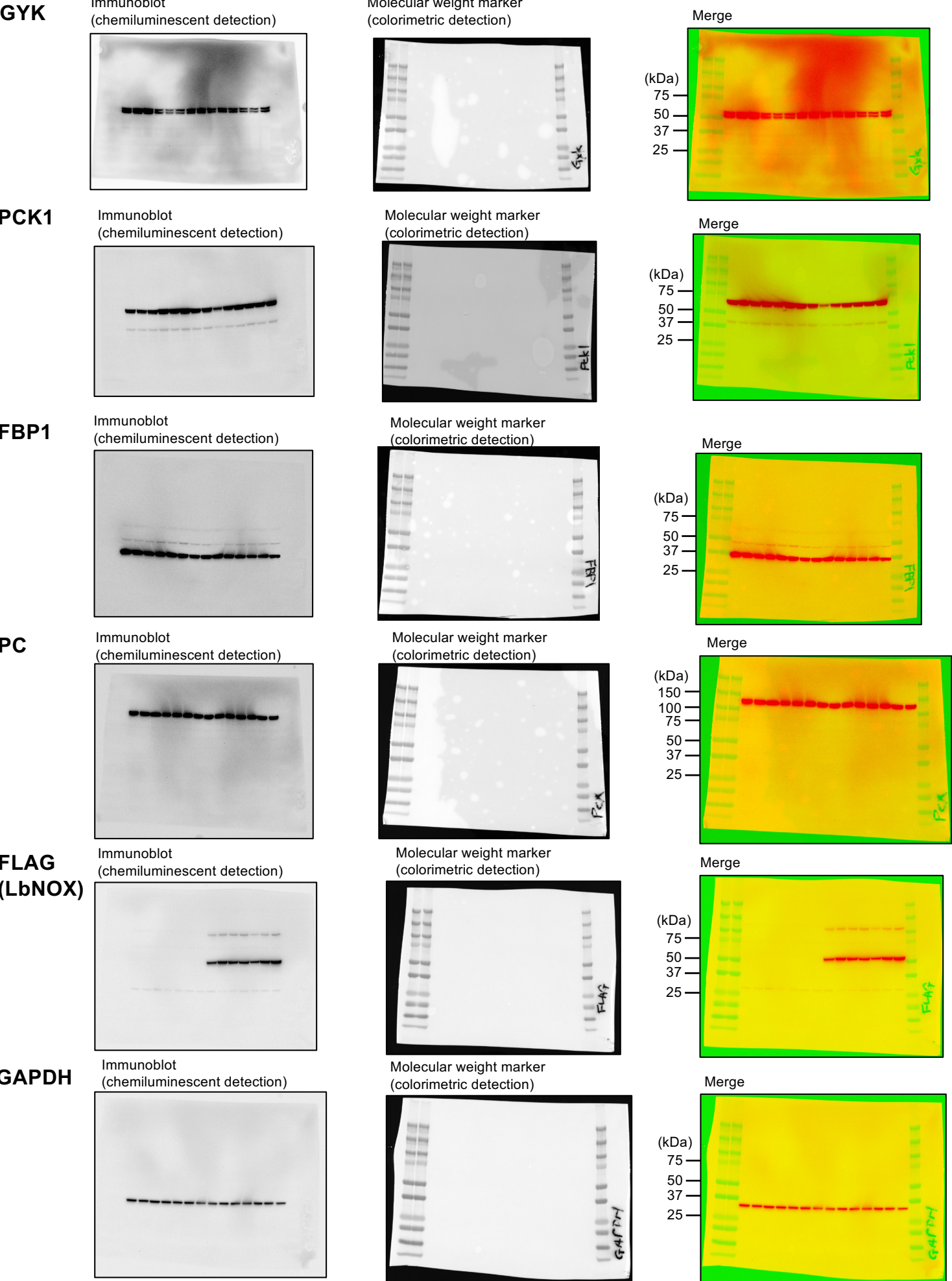

Supplement: Supplementary file 17 — Uncropped western blots. [file 42255_2025_1373_MOESM17_ESM.pdf]
