# Supplementary material for: Redox-dependent liver gluconeogenesis impacts different intensity exercise in mice
Source: Nat Metab. 2025 Sep 18;7(10):1991–2003. doi: 10.1038/s42255-025-01373-z (PMC12552127; doi:10.1038/s42255-025-01373-z)

Extended Data Fig. 2a

PCK1

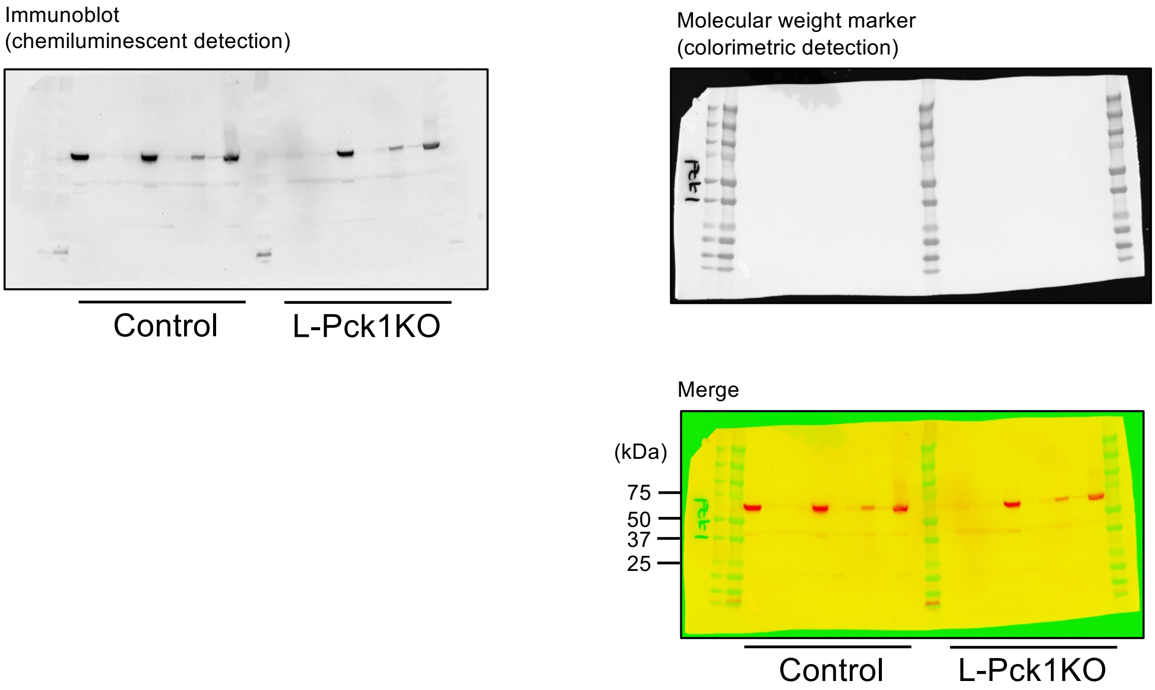

Extended Data Fig. 2b

GYK

Immunoblot
(chemiluminescent detection)

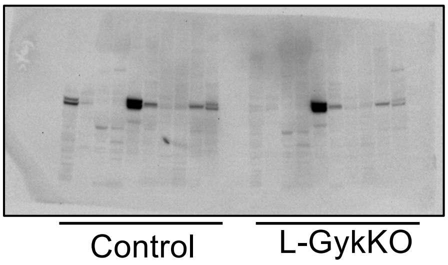

Molecular weight marker
(colorimetric detection)

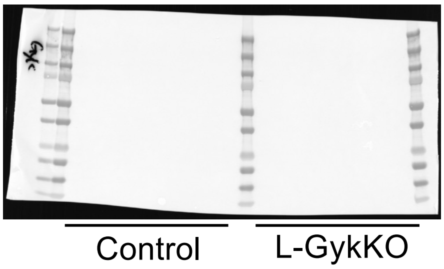

Merge

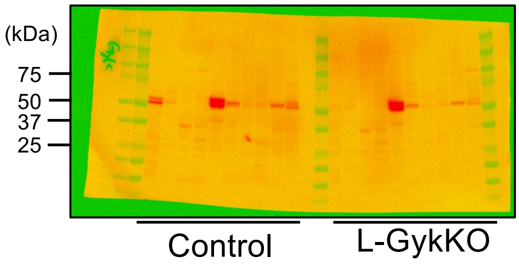

Supplement: Supplementary file 15 — Uncropped western blots. [file 42255_2025_1373_MOESM15_ESM.pdf]
